# Improved wound healing by dual inhibition of miR-146a-5p and miR-29a-3p supports a network action of dysregulated miRNAs in diabetic skin

**DOI:** 10.1007/s00125-025-06522-3

**Published:** 2025-09-03

**Authors:** Marija Petkovic, Ermelindo C. Leal, Anja E. Sørensen, Per T. Jørgensen, Jesper T. Wengel, Rosa R. Jersie-Christensen, Jesper T. Troelsen, Eugenia Carvalho, Louise T. Dalgaard

**Affiliations:** 1 Steno Diabetes Center Copenhagen, Herlev Hospital, Herlev, Denmark; 2 Department of Science and Environment, Roskilde University, Roskilde, Denmark; 3 CNC-UC – Center for Neuroscience and Cell Biology, CIBB – Centre for Innovative Biomedicine and Biotechnology, University of Coimbra, Coimbra, Portugal; 4 Institute for Interdisciplinary Research, University of Coimbra, Coimbra, Portugal; 5 Department of Physics, Chemistry and Pharmacy, University of Southern Denmark, Odense, Denmark; 6 Faculty of Engineering and Science, Aalborg University, Aalborg, Denmark

**Keywords:** Angiogenesis, Collagen, Diabetic wound healing, Extracellular matrix, Inflammation, Laminins, miRNA, Non-coding RNA, Reactive oxygen species

## Abstract

**Aims/hypothesis:**

Upregulation of miR-146a-5p and miR-29-3p is observed in chronic non-healing wounds in diabetes. Their single or combined inhibition's molecular and cellular effects were assessed in human keratinocytes (HaCaT cells) and in vivo using a mouse model of type 1 diabetes.

**Methods:**

As primary outcomes, we screened for proteome changes in HaCaT cells by LC-MS/MS after transfection with miR-146a-5p or miR-29a-3p inhibitors individually or in combination and following stimulation with TNF-α. Moreover, as a secondary outcome, we collected the data and cryopreserved and paraffin-embedded skin biopsies to estimate the tissue response to miRNA inhibition using immunofluorescence and histological analysis. Cryopreserved biopsies were also used for the LC-MS/MS proteome profiling to identify targets and cellular pathways involved in observed tissue changes.

**Results:**

We identified a panel of extracellular matrix proteins, mainly laminins, whose levels changed after transfection with miR-146a-5p or miR-29a-3p inhibitors in HaCaT cells, counteracting TNF-α effects. There was a difference in wound closure rate in vivo between the dual inhibition of miR-146a-5p and miR-29a-3p and scramble controls on day 8 (*p*<0.01) and day 9 (*p*<0.05), although not at day 10. Histological analysis at day 10 shows a loose papillary layer in the scramble inhibition group, indicating incomplete wound closure compared with dual miRNA inhibition. Moreover, the dual action of the inhibitors decreased inflammation at day 3 and day 10 (both *p*<0.001) and reactive oxygen species formation (*p*<0.01) 3 days post wounding, while increasing the angiogenesis on day 3 (*p*<0.01) and day 10 (*p*<0.001). This was consistent with cytoskeletal rearrangements and collagen alterations observed in proteome profiling.

**Conclusions/interpretation:**

These findings demonstrate that dual inhibition of miR-146a-5p and miR-29a-3p in vitro synergises in a bidirectional manner, resulting either in intermediate effects or in cancelling each other’s activity for the levels of specific proteins of basal lamina that impair proliferation and cell motility, compared with the individual inhibitors. Topical supplementation of miR-146a-5p and miR-29a-3p inhibitors to diabetic mouse wounds resulted in a reduction in wound size on days 8 and 9, which correspond to the later stages of healing, but did not lead to complete healing by day 10. However, dual inhibition demonstrates favourable effects on high oxidative stress, elevated inflammation and poor angiogenesis. These effects are superior to single miRNA inhibition, suggesting that combined miRNA inhibition could be a promising therapeutic strategy for diabetic wound healing. Nevertheless, further studies in humans are warranted.

**Graphical Abstract:**

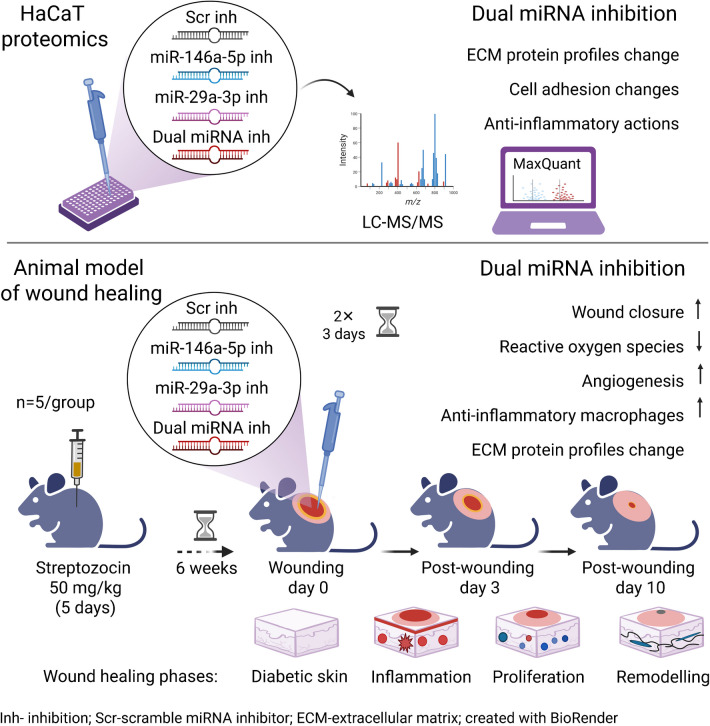

**Supplementary Information:**

The online version contains peer-reviewed but unedited supplementary material available at 10.1007/s00125-025-06522-3.


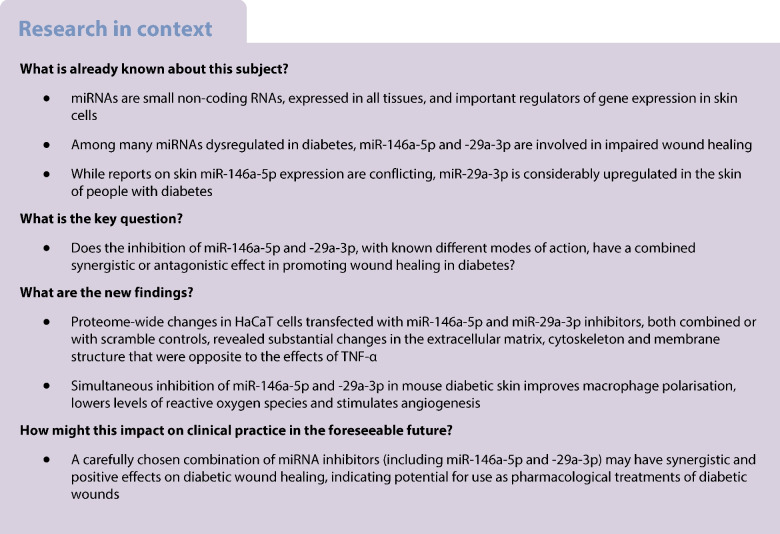


## Introduction

A considerable number of miRNAs expressed in the skin interfere with wound healing-related signalling pathways in diabetic skin [1]. miR-146a-5p and -29a-3p are markedly altered under diabetic conditions [2, 3], and the correction of their dysregulated expression is currently studied for the development of effective strategies to restore the aberrant healing observed in chronic wounds under diabetic conditions [4, 5]. Therapeutic manipulation of inflammatory stage-specific miR-146a-5p levels using cerium oxide nanoparticles was proposed as an approach to repair the poor healing outcome in diabetes [6]. Additionally, it has been shown that inhibition of miR-146a-5p promotes wound healing in human diabetic organ-cultured corneas [7]. The miRNAs belonging to the miR-29 family are recognised as important regulators of extracellular matrix (ECM) remodelling by post-transcriptional regulation of collagen synthesis [8, 9].

While the intricate individual miR-146a-5p- or miR-29a-3p-dependent gene regulation of wound healing has been widely recognised [8–10], the endogenous miRNA crosstalk during wound healing is proposed as an interactive mechanism to fine-tune protein output [11].

The narrow number of experimentally determined miRNA–target interactions has promoted the use of computational predictions to amplify miRNA–target repertoires [12]. In addition, only a few studies have described the potential miRNA–miRNA competitive or cumulative behaviour towards binding to the same target due to completely or partially complementary structures [13]. The computational model designs involve miRNA co-expression or miRNA combinatorial binding [14]. However, the heterogeneous regulatory effects of both miRNAs and mRNAs exerted in a cell-type-specific manner may differ in silico and in vivo, and thus their interaction cannot be accurately described using prediction algorithms [15].

Despite substantial advances in preclinical research, the field of miRNA-based therapeutics remains in its early stages, with only a few candidate compounds progressing to clinical development and other candidate compounds undergoing early termination due to toxicity issues or research costs [16].

Diabetic wound healing is characterised by impaired cell motility and proliferation [8]; increases in proinflammatory cells with consequent increases in the inflammatory state, proteolytic enzymes and reactive oxygen species (ROS) levels [17]; inadequate vascularisation network [18]; and, finally, faulty tissue remodelling [19]. Given the complexity of diabetic wound healing, a comprehensive understanding of multiple miRNA roles in wound repair could be advanced by mapping the cellular crosstalk in dynamic miRNA regulation using in vitro and in vivo models.

Considering their roles in diabetic wound healing impairments, we hypothesised that dual inhibition of miR-146a-5p and -29a-3p, both upregulated in diabetes, could be beneficial for diabetic wound healing. Therefore, the effects of inhibition of miR-146a-5p and -29a-3p were evaluated in vitro and in vivo, using a HaCaT keratinocyte model and a recognised mouse model of diabetic wound healing [20].

## Methods

### HaCaT cell model

Immortalised keratinocytes (HaCaT cells) maintained in DMEM and mycoplasma free, were stimulated with TNF-α (10 ng/ml) or transfected with 25 pmol locked nucleic acid (LNA)-spiked fully phosphorothioate-modified antisense miR-146a-5p (5′-TGgaauucAgTuCucA-3′) and miR-29a-3p (5′-AuuTcaGauGguGcuA-3′) oligos (LNA bases are written in capital letters while 2′-O-methyl RNA bases are in small letters) and a scramble (Scr) control oligo (5′-CaaTagGguCaaGauT-3′), individually or in combination (12.5 pmol of each inhibitor), and cell lysates were harvested for proteomic analysis. See the electronic supplementary material (ESM) Methods.

### Animal type 1 diabetes model of wound healing impairments

Male C57BL6 mice (25–30 g, Charles River, Barcelona, Spain) were housed in a specific pathogen-free barrier facility, on a 12 h light/12 h dark cycle at 22–24°C with access to water and chow diet ad libitum. All animal studies were under the European Community law for Experimental Animal studies (86/609/CEE and 2007/526/CE), approved by the Institutional and Ethical Board at the University of Coimbra and by the Governmental (Directorate-General for Food and Veterinary of the Portuguese Ministry of Agriculture) Research Ethical Boards, and following the Guide for the Care and Use of Laboratory Animals. Mice that met inclusion criteria of having the glucose levels >14 mmol/l , were used for wound healing studies 6 weeks after streptozocin (STZ) (50 mg/kg) (Sigma-Aldrich, St Louis, MO, USA) diabetes induction, and skin collection from normoglycaemic and diabetic mice, or from mice randomly allocated to topical treatments with miR-146a-5p or miR-29a-3p inhibitors individually (2.5 nmol) or in combination (1.25 nmol of each inhibitor), or a Scr inhibitor (2.5 nmol), twice daily for the first 3 days (ESM Methods).

### Wound healing kinetics

Wound closure progression was traced every day with acetate paper and digitally recorded on days 0, 3, 7 and 10. The researcher performing wound healing studies was blinded to the treatment allocation during assessments of wound areas obtained from acetate tracing. The wound diameters were quantified with ImageJ2 (NIH, USA). Mixed methods modelling was used to assess the effect of treatment over time, presented as mean ± SD, one-way ANOVA, with Tukey's multiple comparison test, using R-studio (version 2024.12.0+467, https://posit.co/downloads/) and the nlme, mice and emmeans packages (https://cran.r-project.org/).

### RNA extraction and RT-qPCR

Skin tissue (~50 mg) was homogenised in TRI Reagent (Sigma-Aldrich). RNA concentration and purity were assessed using the Nanodrop ND-1000 spectrophotometer (Thermo Fisher Scientific, Waltham, MA, USA).

miRNAs were detected using reverse transcription with quantitative PCR (RT-qPCR) (ESM Methods). Oligonucleotides used for RT-qPCR are shown in ESM Table 1.

### Proteomic analyses

Whole-cell lysates of HaCaT keratinocytes or skin biopsies (~20 mg) were prepared using a guanidine lysis buffer [21]. Following enzymatic digestion, peptides (5 µg) were subjected to high-resolution mass spectrometry LC-MS/MS analysis. Raw data were processed with MaxQuant v1.6.12.0 (https://www.maxquant.org/) with default settings including label-free quantification (LFQ) and searched against UniProtKB *Homo sapiens* or *Mus musculus* reference proteomes. LFQ values were used for data interpretation and initial statistical analysis employed Perseus software 1.6.15.0 (https://www.maxquant.org/) to identify differentially expressed proteins (ESM Methods).

### Reporter gene analyses

The laminin γ2 chain (LAMC2; encoded by *LAMC2*) promoter and enhancer (luciferase-coupled) plasmid construct was used for reporter gene analysis [22]. In two individual experiments, HaCaT cells were transfected with 450 ng of LAMC2/luciferase construct and treated with TNF-α (10 ng/ml) or miRNA inhibitors (ESM Methods). After 24 h, cells were harvested and lysed, and luciferase activity was determined using the Dual Light system (Tropix; Perkin Elmer) using a GloMax96 instrument (Promega).

### Immunofluorescence

The M1/M2 macrophage ratio and the rate of neovascularisation were measured using immunofluorescence. Primary antibodies: rabbit polyclonal anti-CD68 (ab125212, Abcam, Cambridge, UK), rat monoclonal anti-TNF-α (MCA1488, AbD Serotec, Algés, Portugal), rat monoclonal anti-CD206 (MR5D3) (sc-58987, Santa Cruz Biotechnology, Santa Cruz, USA) and rat monoclonal anti-CD31 (PECAM-1) (CBL1337, Merck Millipore, Darmstadt, Germany) antibodies were diluted 1:100. Secondary antibodies: anti-rat (conjugated to Alexa Fluor 568, Invitrogen) and anti-rabbit (Alexa Fluor 468-conjugated, Invitrogen), diluted 1:500 (ESM Methods). Fluorescence images were obtained with a confocal microscope (Zeiss LSM 510 Meta) and ZEN (blue edition) 2.1 SP3 software (Zeiss Microscopy).

### Fluorescence analysis of dihydroethidium for ROS detection

Dihydroethidium (DHE) staining was performed on 30 μm optimal cutting temperature (OCT)-embedded frozen sections following the manufacturer’s instructions (Thermo Fisher Scientific) (ESM Methods). Fluorescence images were acquired with a Zeiss LSM 510 Meta confocal microscope.

### Histological analyses

Formalin-fixed and paraffin-embedded skin sections (3 µm) were stained using H&E (Sigma-Aldrich) and Masson–Goldner Trichrome (Carl Roth, Germany), according to the manufacturer protocols (ESM Methods). Images were acquired using a Zeiss Axiovert Imager wide-field microscope.

### Western blots

Skin tissue was homogenised in RIPA lysis buffer. Then, 30 µg of total protein was resolved on a 7.5% SDS-PAGE gel and transferred to a PVDF membrane, and probed with primary rabbit anti-IL receptor-associated kinase 1 (IRAK1) (ADI-905-709-100, Enzo Life Sciences, dilution 1:500), or primary rabbit anti-PI3 kinase antibody, p85 polyclonal (06-195, Millipore, diluted 1:1000), followed by secondary goat anti-rabbit IgG-HRP (31402, Thermo Fisher Scientific, diluted 1:5000). Membranes were revealed with enhanced chemiluminescence substrate (Pierce, Thermo Fisher Scientific) and visualised with a ChemiDoc Touch Gel Imaging System (Bio-Rad Laboratories, Amadora, Portugal). Data were normalised to goat anti-β-Actin (sc-1616, Santa Cruz Biotechnology, diluted 1:1000). Densiometric analyses were performed in Image Lab Software version 6.0 (Bio-Rad Laboratories) (ESM Methods).

### Statistical analyses

Statistical analyses of differences between the groups were performed using a two-sided *t* test with corrections for multiple tests, or using one-way or two-way ANOVA followed by Tukey’s post hoc test in GraphPad Prism 10.1.0 or permutation-based false discovery rate (FDR) correction for multiple hypothesis testing in Perseus 1.6.15.0 (proteomic studies). Data were presented as mean ± SD. The *p* values lower than 0.05 were considered statistically significant. Detailed descriptions of statistical analyses from replica experiments and experimental data handling are provided in the ESM Methods.

## Results

### Differential regulation of miR-146a and miR-29a by wounding in diabetic mouse skin

**Fig. 1:**
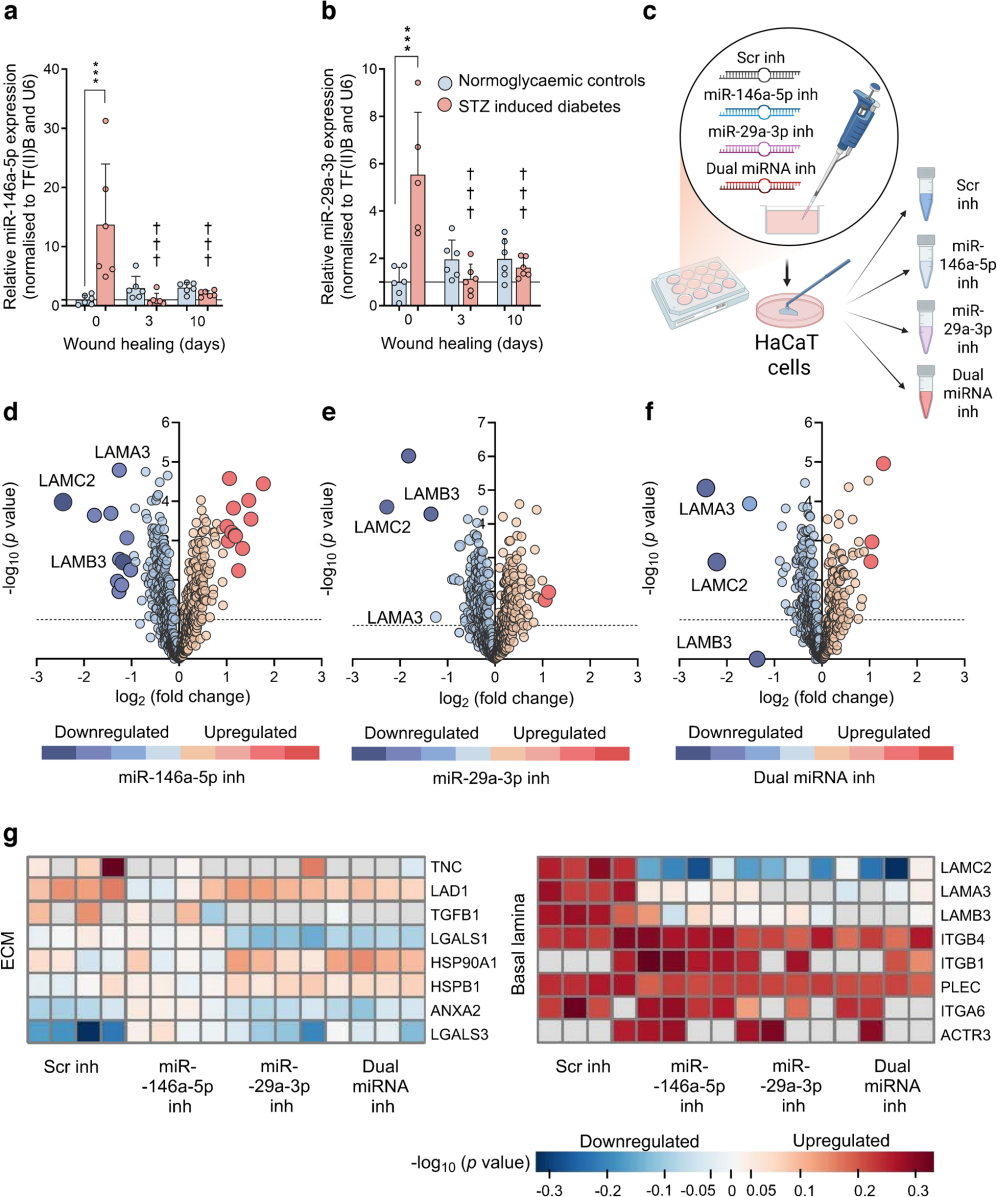
Relative expression levels of (**a**) miR-146a-5p and (**b**) miR-29a-3p were determined in normoglycaemic and STZ-induced diabetic mouse skin before wounding, and at days 3 and 10 post wounding (*n*=6 animals/group), by RT-qPCR. ***p<0.001 (magnitude of miR-146a-5p and -29a-3p expression levels vs normoglycaemic group), ^†††^*p*<0.001 (miR-146a-5p and -29a-3p expression levels in diabetic group pre and 3 and 10 days post wounding vs before wounding) (data presented as mean ± SD); U6 small nuclear RNA (U6) and, transcription factor IIB (TF(II)B) were used as internal positive controls. (**c**) HaCaT proteomics workflow. (**d**–**f**) Volcano plots showing the distribution of proteins identified after targeted individual or combinatory actions of miR-146a-5p and miR-29a-3p inhibitors in vitro (*N*=16, *n*=4 wells/group), respectively. Protein levels are shown according to *p* value and fold change (downregulation level in blue and upregulation level in red, with a colour-coded degree of difference). (**g**) Heatmaps showing the effects of differential and synergistic actions of miRNA inhibitors on HaCaT cell lysate composition of proteins, belonging to the ECM and basal lamina (blue indicates downregulation, red indicates upregulation). Fig. 1c was created in BioRender. Petkovic, M. (2025) https://BioRender.com/i77w902. Inh, inhibitor; TGFB1, TGF-β1; LGALS1, galectin-1; HSP90A1, putative heat shock protein 90 α1; HSPB1, putative heat shock protein β1; ANXA2, annexin A2; LGALS3, galectin-3; PLEC, plectin; ACTR3, alpha-centractin-3

Differential expression of selected miRNAs in normoglycaemic and STZ-induced diabetic mouse skin was determined with RT-qPCR (Fig. 1). miR-146a-5p levels in unwounded skin were increased by diabetes (13.7 ± 10.3-fold) when compared with controls (*p*<0.001). At the same time, the levels were markedly suppressed 3 days (*p*<0.001) and 10 days post wounding (*p*<0.001) in diabetic skin (Fig. 1a). miR-29a-3p levels were also markedly elevated (5.5 ± 2.6-fold) in diabetic skin relative to normoglycaemic controls (*p*<0.001). In contrast, their levels were suppressed after wounding in diabetic mice at day 3 (*p*<0.001) as well as at day 10 (*p*<0.001) after wound induction (Fig. 1b).

### miR-146a and miR-29a-3p inhibitors remodel the basement membrane protein architecture

**Fig. 2:**
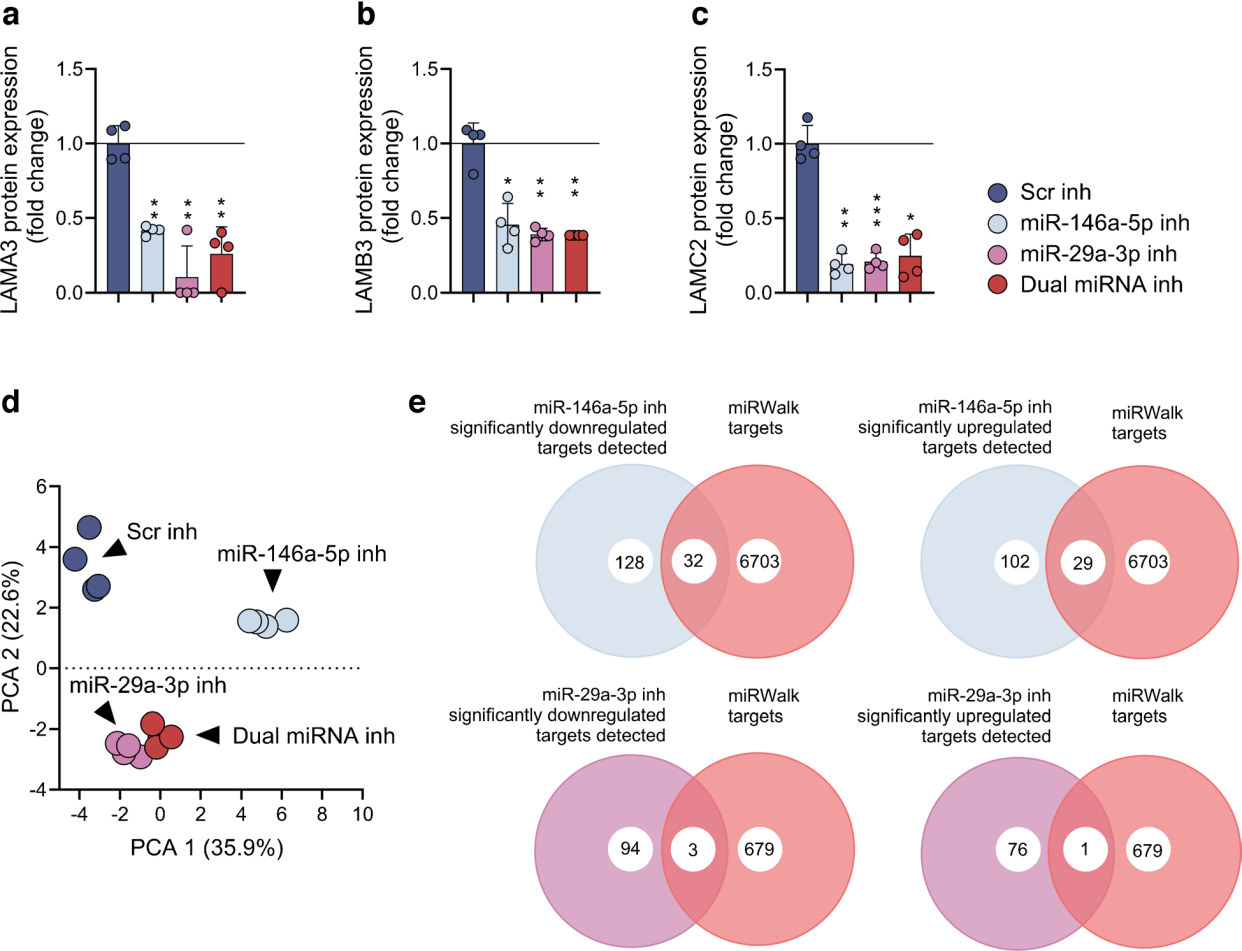
Comparison of the HaCaT cell proteome profile after miR-146a-5p and miR-29a-3p inhibition. (**a**–**c**) Expression levels of laminins changed by treatments, and regulated by either individual or combinatory miRNA inhibitor actions (*N*=16, *n*=4 wells/group). Data presented as mean ± SD. (**d**) PCA of HaCaT cell proteome exposed to individual and dual miR-146a-5p and miR-29a-3p inhibition. (**e**) Venn diagrams illustrate the overlap of proteins up- and downregulated by miR-146a-5p and miR-29a-3p inhibition (predicted targets described in the miRWalk database are shown in ESM Table 2, while the complete list of those markedly up- or downregulated by miRNA inhibition is in ESM Table 3). *p<0.05, **p<0.01, ***p<0.001 vs Scr inhibitor. Inh, inhibitor

A proteome-wide analysis of whole-cell lysates treated with a Scr control, miR-146a-5p or miR-29a-3p inhibitors, or combined inhibition (Fig. 1c), robustly detected 1450 proteins. The most striking downregulated protein expressions were observed for laminin α3 chain (LAMA3), laminin β3 chain (LAMB3) and LAMC2. miR-146a-5p and miR-29a-3p inhibition strongly repressed LAMC2 expression, together with the other laminin 5 complex (LM332) subunits (Figs 1d–f, 2a–c).

The direction of change of integrin α6 (ITGA6), integrin β1 (ITGB1) and integrin β4 (ITGB4), particularly the ITGB4/ITGA6 laminin receptor, was opposite to the expression of their ligands, of the LAM332 family (Fig. 1g). Moreover, tenascin C (TNC), a ligand for integrin α8 (ITGA8)/ITGB1 and integrin α9 (ITGA9)/ITGB1, was downregulated by miR-146a-5p inhibitor and as a part of the dual treatment, while miR-29a-3p inhibition and Scr control maintained higher levels of TNC (Fig. 1g). Ladinin 1 (LAD1) was partially downregulated by individual miR-146a-5p inhibition while the other inhibitory treatments had the opposite effect. Furthermore, TGF-β1 (TGFB1 ) was negatively regulated by miR-29a-3p inhibition alone or in dual treatment compared with the miR-146a-5p individual treatment (Fig. 1g).

Principal component analysis (PCA) separated the four treatments into clusters of Scr, miR-146a-5p and miR-29a-3p inhibitors and dual inhibition, with the dual inhibitor treatment positioned between the effects of inhibiting miR-146a-5p and miR-29a-3p, although closer to the miR-29a-3p inhibitor cluster (Fig. 2d). A functional correlation of miRNA inhibition up/downregulated proteins with predicted targets is described in the miRWalk database (ESM Table 2) [23]. The miRWalk database identifies 6703 proteins as predicted targets of miR-146a-5p (ESM Table 2a). Of 128 miR-146a-5p inhibitor-downregulated proteins, 32 were predicted targets, while 29 predicted targets were upregulated proteins (Fig. 2e). Furthermore, 679 proteins were predicted as targets of miR-29a-3p (ESM Table 2b). We report 94 miR-29a-3p inhibitor-downregulated proteins, with three proteins being miR-29a-3p targets, while of 76 upregulated proteins, only one is a known target of miR-29a-3p. An overview of the up/downregulated proteins is presented in Fig. 2e, while the full list is in ESM Table 3.

The levels of IRAK1, a known miR-146a-5p target, increased with dual miRNA inhibition (2.5 ± 0.9-fold) compared with Scr inhibition (0.8 ± 0.6-fold, *p*<0.01) during the inflammatory phase at 3 days post wounding (ESM Fig. 1a, b). IRAK1 protein levels were lowered 10 days post wounding when suppressing both miRNAs (0.5 ± 0.3-fold, *p*<0.001) (ESM Fig. 1a, b). Furthermore, we probed for the miR-29a-3p target p85, observing increased protein expression in combined inhibition with miR-146a-5p (2.9 ± 0.4-fold) at 3 days post wounding, compared with Scr inhibition (2.2-fold ± 0.3, NS). Moreover, p85 remained increased with the combined treatment (2.1-fold ± 0.7) compared with miR-29a-3p (0.9-fold ± 0.3, *p*<0.01) alone and Scr inhibition (0.9-fold ± 0.2, *p*<0.001) at 10 days post wounding (ESM Fig. 1c,d).

### Opposing actions of TNF-α and miRNA inhibition in keratinocytes

**Fig. 3:**
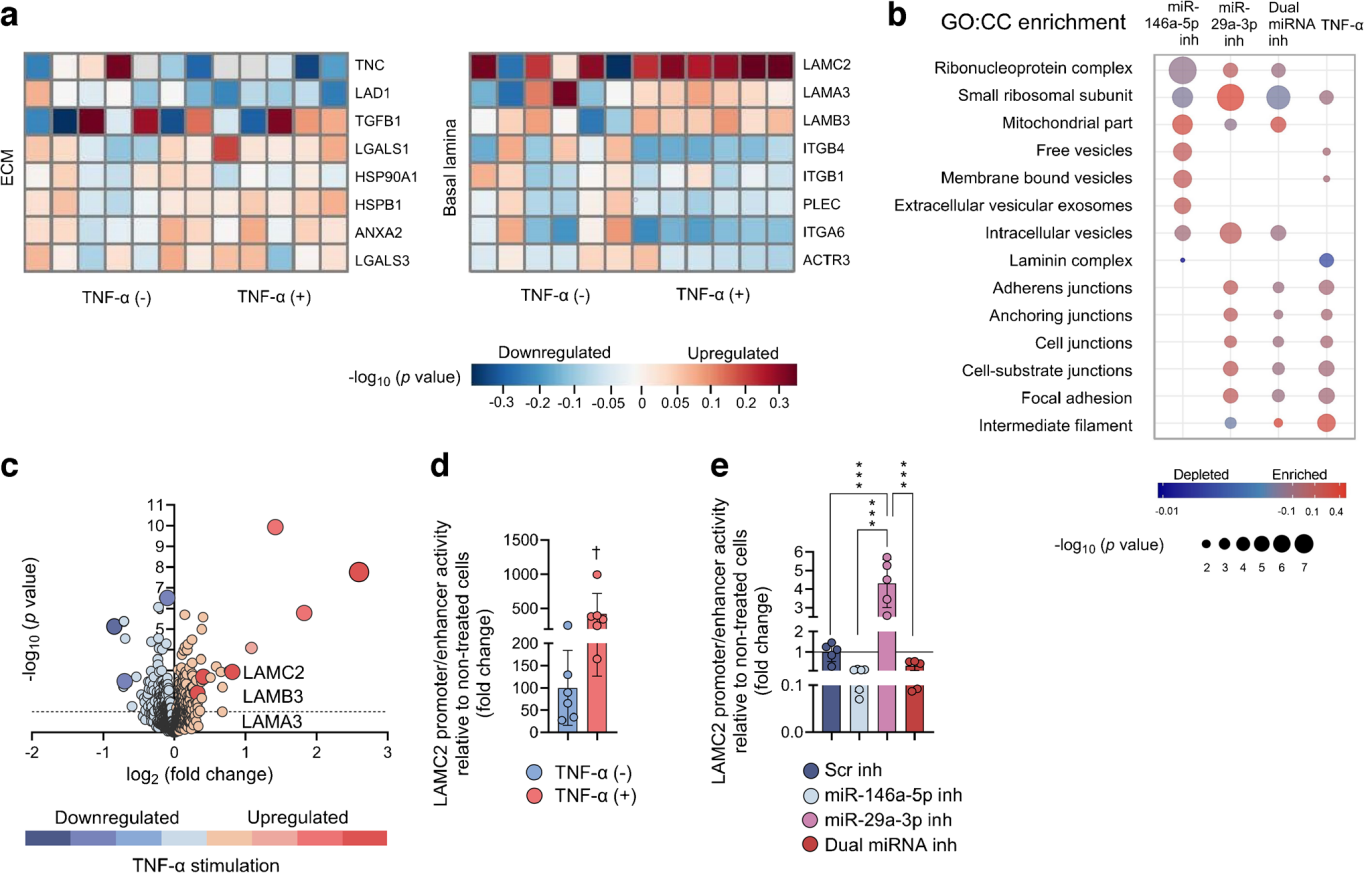
LC-MS/MS-based proteome profiling of TNF-α-regulated proteins in HaCaT keratinocytes. (**a**) Heatmaps illustrate the change of basal membrane laminins, and ECM protein families, regulated in response to TNF-α stimulation (*N*=12, *n*=6 wells/group). (**b**) GO:CC category enrichment for cell–cell adhesion, and ECM integrity components regulated by individual and dual inhibition of miR-146a-5p and miR-29a-3p, and by TNF-α (ESM Table 4). (**c**) TNF-α stimulation elevates the expression of laminins. (**d**) LAMC2 promoter/enhancer activity of HaCaT cells stimulated with TNF-α (data presented as mean ± SD, expressed as percentage relative to the TNF-α (–) control). (**e**) LAMC2 promoter/enhancer activity of HaCaT cells transfected with the miR-146a-5p inhibitor, the miR-29a-3p inhibitor, the combination of the two inhibitors or Scr inhibitor (data presented as mean ± SD, expressed as fold change relative to Scr-inh condition). Experiments for LAMC2 promoter activities were performed in sextuplicate for each promoter, and promoter activity was normalised to the protein content in the cell extracts. ^†^*p*<0.05 vs TNF-α (–), ***p<0.001 (comparisons as shown). Inh, inhibitor, TGFB1, TGF-β1; LGALS1, galectin-1; HSP90A1, putative heat shock protein 90 α1; HSPB1, putative heat shock protein β1; ANXA2, annexin A2; LGALS3, galectin-3; PLEC, plectin; ACTR3, alpha-centractin-3

The actions of miR-146a-5p and miR-29a-3p inhibitors were assessed on the keratinocyte proteome and compared with those of TNF-α-stimulated HaCaT cells (ESM Table 4). A compelling number of the basal lamina and ECM proteins exposed to dual inhibitor treatment changed expression in the opposite directions compared with those of TNF-α, as shown in Fig. 3a (detailed description in ESM Figs 2, 3). Interestingly, the enrichments of Gene Ontology Cellular Component (GO:CC) categories related to RNA processing were attributed to miRNA-146a-5p treatment over TNF-α activation. Moreover, extracellular/intracellular vesicle-related enrichment with miR-146a-5p inhibition and their depletion by miR-29a-3p inhibition suggests discriminative actions of the two miRNA inhibitors. Cellular adhesion components were completely depleted by miR-146a-5p inhibitor treatment (Fig. 3b). GO:CC enrichments of the combined inhibitor treatment were generally intermediate, in comparison with the effects of the two single miRNA inhibitor treatments (Fig. 3b).

LAMA3, LAMB3 and LAMC2 stood out as markedly upregulated by TNF-α-induced inflammation (Fig. 3c, ESM Fig. 2). As LAMC2 is a predicted target of miR-29a-3p, we employed a luciferase reporter construct containing both the proximal promoter and a TNF-α-responsive enhancer to measure the transcriptional response of LAMC2 to miRNA inhibitor treatment, or to TNF-α. TNF-α increased LAMC2 promoter/enhancer activity by fourfold compared with TNF-α non-treated HaCaT cells (Fig. 3d).

Interestingly, miR-29a-3p inhibition increased the LAMC2 promoter/enhancer activity, relative to the Scr, unlike the miR-146a-5p inhibitor/LAMC2 co-transfected cells, while the dual inhibition resulted in intermediate activity of the LAMC2 promoter/enhancer (Fig. 3e), indicating that the miRNA inhibitors preferentially act via a transcriptional mechanism in regulating LAMC2.

### Dual inhibition improves overall wound healing and decreases inflammation in the wound bed of diabetic animals

**Fig. 4:**
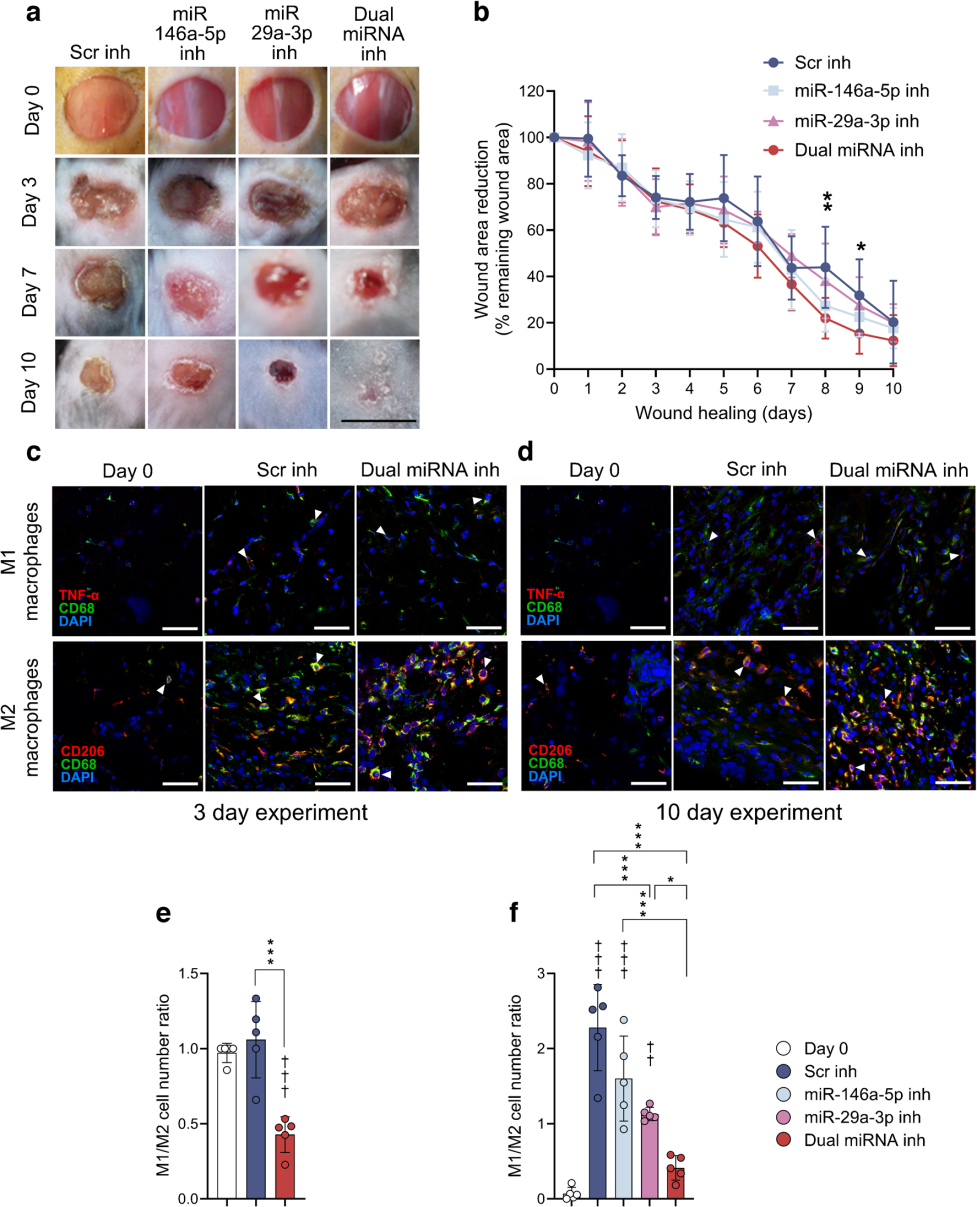
Topical treatment with miR-46a-5p and miR-29a-3p inhibitors facilitates wound area reduction in diabetic mice. Dermal treatments were: 2.5 nmol Scr inhibition (negative control), 2.5 nmol miR-146a-5p inhibitor, miR-29a-3p inhibitor or the combination of 2.5 nmol miR-146a-5p/-29a-3p inhibitors (1.25 nmol each inhibitor). (**a**) Representative photos taken at days 0, 3, 7 and 10 post wounding. (**b**) Wounds are presented as remaining wound area percentage of the initial wound area of 6 mm. **p<0.01 (dual miRNA inhibition vs Scr inhibitor on day 8) and *p<0.05 (miR-146a-5p inhibitor vs Scr inhibitor on day 9). (**c**, **d**) Immunofluorescence analysis of the M1 (proinflammatory) and M2 (anti-inflammatory) macrophages in diabetic wounds, treated with Scr inhibitor, or miR-146a-5p/-29a-3p inhibitors individually, or the combination. (**c**) Representative fluorescence images of skin sections were obtained 3 days post wounding (proinflammatory on the top and anti-inflammatory on the bottom panel). (**d**) Representative images from confocal microscopy showing the M1 (top panel) and M2 (bottom panel) macrophages in skin collected 10 days post wounding. (**e**) The ratio of M1 proinflammatory to M2 anti-inflammatory macrophages quantified 3 days post wounding and calculated as the mean of the number of CD68-positive cells co-stained with TNF-α divided by the mean of the number of cells stained with CD68, and co-stained with CD206 (data presented as mean ± SD). (**f**) The M1 to M2 ratio in skin sections 10 days post surgical collection of skin as a measure of inflammatory environment (data presented as mean ± SD). *p<0.05, ***p<0.001 (comparisons as shown in **e, f**); ^††^*p*<0.01, ^†††^*p*<0.001 vs macrophage pre-wounding baseline numbers (day 0). (**a**) Scale bar, 6 mm (the initial wound area); (**c**, **d**) scale bars, 50 µm; magnification ×400 with immersion oil. Inh, inhibitor

The dual miR-146a-5p and miR-29a-3p inhibitors reduced a wound area to 22.0 ± 8.8% (day 8, *p*<0.01) and 15.4 ± 8.7% (day 9, *p*<0.05) of initial wound size, compared with the Scr control (44 ± 17.5% and 31.9 ± 15.5%, respectively) (Fig. 4a,b). Although we were unable to confirm the difference in wound size on day 10, histological specimens prepared after scab removal revealed persistent subepidermal oedema in the Scr control treatment, indicating incomplete tissue repair, in contrast to the dual inhibitor-treated skin (ESM Fig. 4). Additionally, a notable influx of mononuclear cells (predominantly macrophages) was infiltrating the dermis in response to the miR-146a-5p inhibitor or the combined treatment (ESM Fig. 4). This observation was further validated by immunofluorescent labelling of CD68 markers, co-stained with TNF-α for M1 (proinflammatory) and with CD206 for M2 (anti-inflammatory) macrophages (Fig. 4c, d, ESM Fig. 5). The M1/M2 ratio was decreased by the dual inhibition treatment (0.5 ± 0.1) compared with the Scr (1.1 ± 0.3, *p*<0.001) at 3 days post wounding (Fig. 4e, ESM Fig. 5), and this was even more pronounced at 10 days post wounding: dual miRNA inhibition (0.4 ± 0.2, *p*<0.001), and miR-29a-3p inhibition (1.1 ± 0.1, *p*<0.05) vs Scr inhibition (2.3 ± 0.6), while there was no difference with miR-146a-5p inhibition (1.6 ± 0.6, NS) (Fig. 4f, ESM Fig. 5).

### Effects of dual miRNA inhibition on wound superoxide generation

**Fig. 5:**
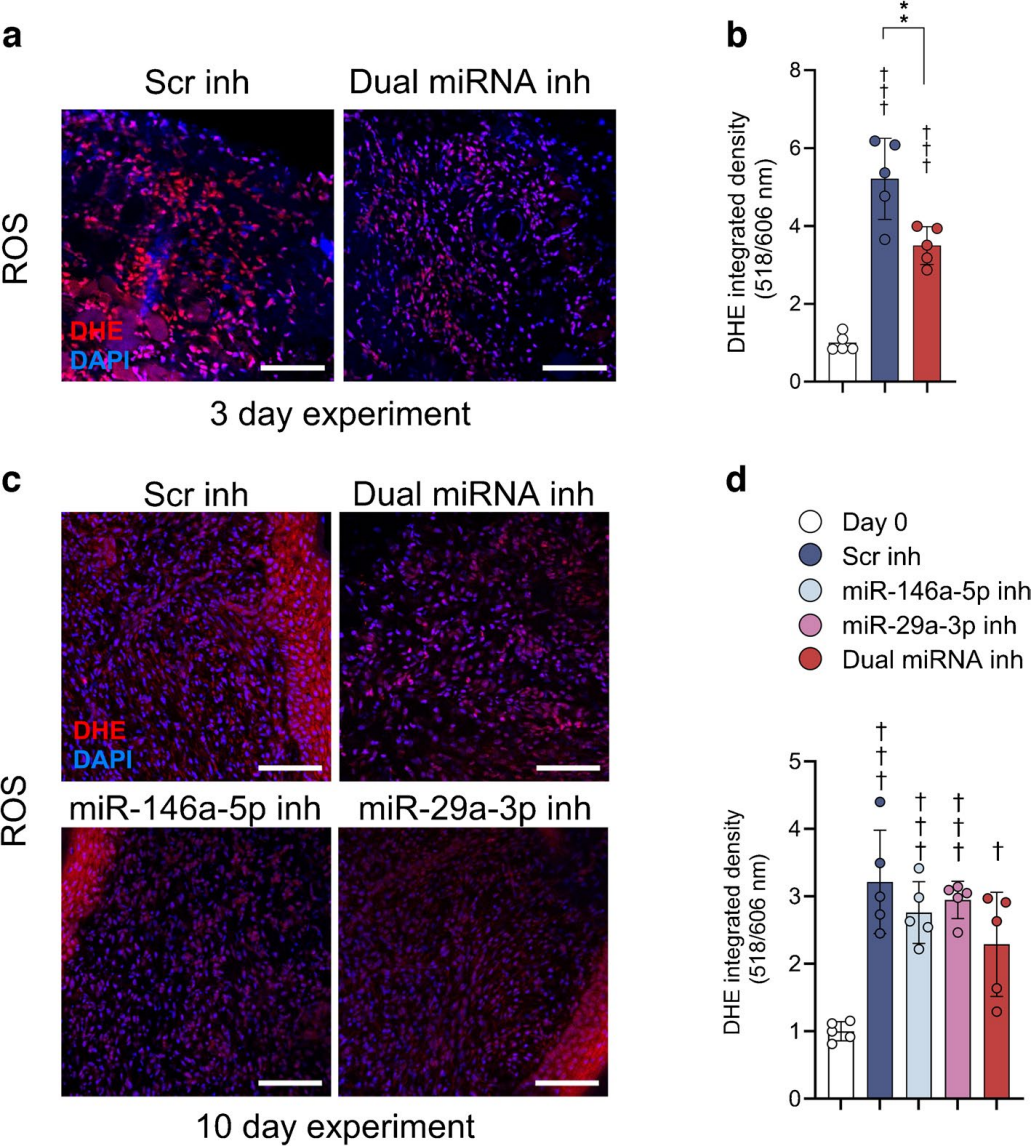
ROS production in murine wound skin measured by DHE dye-based detection after single or combined miR-146a-5p and -29a-3p inhibition. (**a**) Representative microscopy images of DHE-stained wound skin sections harvested after 3 day treatments with Scr inhibitor (2.5 nmol) or inhibitors of miR-146a-5p and miR-29a-3p in combination (1.25 nmol of each inhibitor). (**b**) Quantification of ROS formation 3 days after the wounding was achieved by measuring mean integrated density grey values of DHE signal (*n*=5 animals per group; six pictures/five random square fields per picture and per sample analysed). (**c**) Representative pictures from DHE-stained wound skin sections collected 10 days post wounding after 3 day treatments with Scr inhibitor (2.5 nmol) or inhibitors of miR-146a-5p and miR-29a-3p in combination (1.25 nmol of each inhibitor). (**d**) Quantification of ROS formation by measuring DHE signal 10 days after the wounding (*n*=5 animals per group; six pictures/five random square fields per picture and per sample analysed). Data in (**b**, **d**) are presented as mean ± SD. **p<0.01 (miR-146a-5p/-29a-3p dual inhibition changed DHE signal relative to Scr) and ^†^*p*<0.05, ^†††^*p*<0.001 vs pre-wounding baseline values. (**a, c**) Scale bars, 50 µm; magnification ×200. Inh, inhibitor

The impact of the dual miRNA inhibition on ROS generation during wound healing was estimated using DHE as a superoxide probe. At day 3 post wound induction, superoxide generation from wound sections was decreased by dual inhibitor treatment (3.5 ± 0.5-fold relative to baseline) compared with Scr control (5.2 ± 1.0-fold, *p*<0.01) (Fig. 5a, b). In contrast, at day 10 post wounding there was no effect on superoxide generation by inhibition of miR-146a-5p or miR-29a-3p, or the combination of both (Fig. 5c, d).

### Improved blood vessel/tissue stability by dual miRNA inhibition

**Fig. 6:**
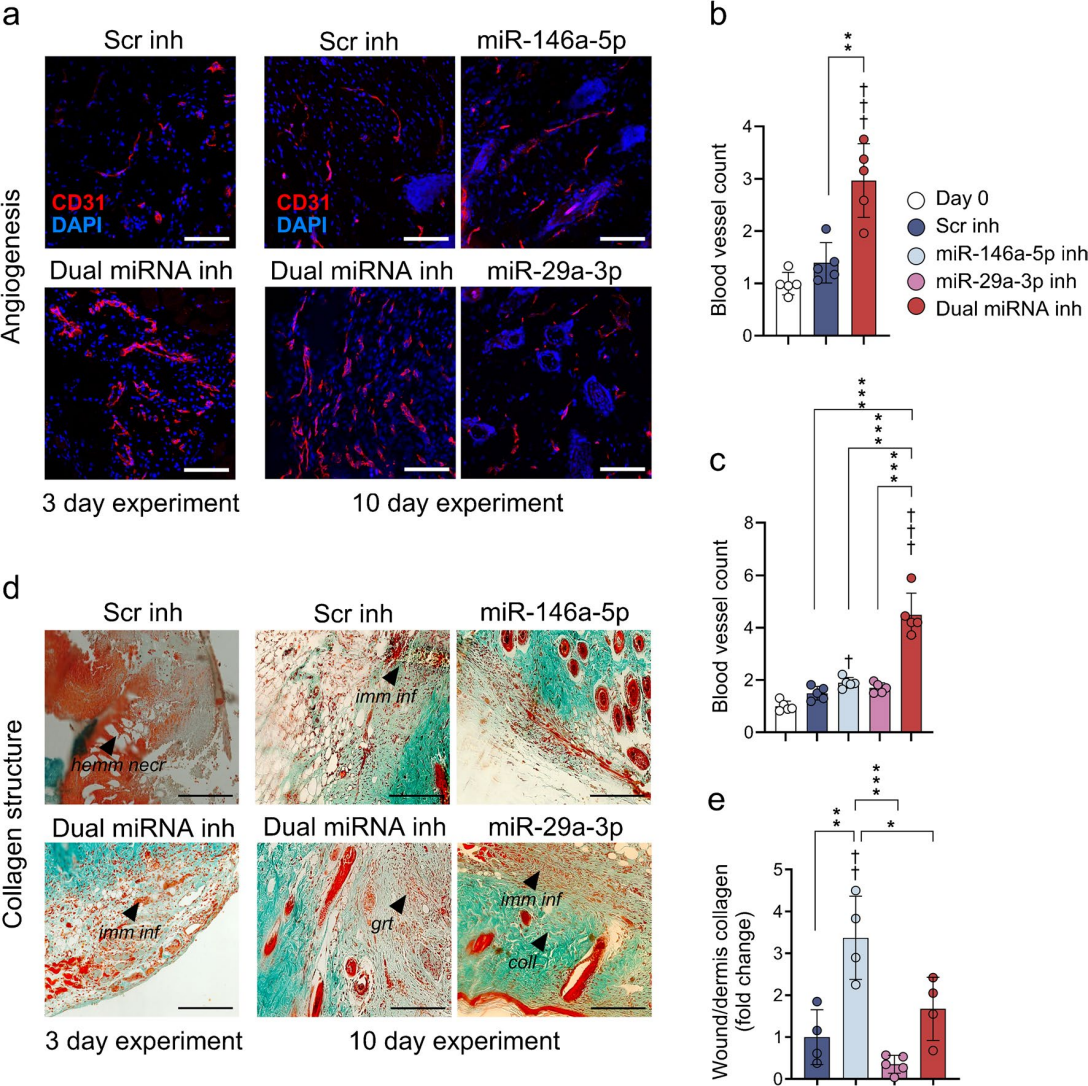
Histological analysis of angiogenesis and collagen deposition in diabetic mouse skin. (**a**) Representative fluorescence pictures of day 3 and day 10 biopsies treated with Scr inhibition (2.5 nmol) or with miR-146a-5p and miR-29a-3p inhibitors (2.5 nmol) individually or in combination (1.25 nmol each). (**b**) The average vessel numbers after 3 days were calculated as the fold change in the number of branches stained in red with CD31 marker for angiogenesis (*n*=5 animals per group; wounds in duplicate and five images taken for sample). (**c**) Fold change of number of newly formed blood vessels in skin harvested 10 days post wounding. (**d**) Representative microscopy images of Masson's trichrome staining of diabetic skin harvested 10 days after wounding following the treatment with Scr inhibitor (negative control) or with 2.5 nmol of miR-146a-5p and -29a-3p inhibitors, separately or in combination. Hemm necr, signs of necrosis with haemorrhage; imm inf, immune cell-enriched tissue and highly organised collagen fibres resemble the structure of healthy scar in miRNA combination or miR-29a treatment; grt, granulation tissue; coll, well-structured collagen bundles. (**e**) Quantification of collagen structures measured as the ratio between the dermal collagen and the collagen in the wound area. Data in (**b**, **c**, **e**) are presented as mean ± SD. *p<0.05, **p<0.01, ***p<0.001 (comparisons as shown) and ^†^*p*<0.05, ^††^*p*<0.01, ^†††^*p*<0.001 vs pre-wounding baseline values. (**a**, **d**) Scale bars, 50 µm; magnification ×200 (*n*=4–5 animals per group; five images for CD31-stained sections and ten images for Masson's trichrome-stained sections taken per sample). Inh, inhibitor

The number of CD31-positive blood vessels was consistently higher at 3 days post wounding by the dual inhibition treatment (3.0 ± 0.7-fold relative to baseline) compared with the Scr control (1.4 ± 0.4-fold, *p*<0.01) (Fig. 6a, b). Similar results were obtained at 10 days post wounding with the dual inhibition treatment (4.5 ± 0.8-fold) when compared with the single inhibition of miR-146a-5p (1.9 ± 0.2-fold, *p*<0.001) or miR-29a-3p (1.7 ± 0.2-fold, *p*<0.001), or Scr control (1.5 ± 0.3-fold, *p*<0.001) (Fig. 6c, d). Furthermore, the Masson's trichrome staining showed increased ECM deposition with the miR-146a-5p inhibition (3.4 ± 1.0-fold, *p*<0.01) while the dual inhibition (1.8 ± 0.7-fold, NS) or the miR-29a-3p inhibition (0.4 ± 0.2-fold, NS) showed no ECM improvements relative to Scr control (Fig. 6d, e), consistent with the upregulation of ECM components in HaCaT cells measured by proteomics.

### Cell transfection-induced changes resemble inhibitor actions observed in diabetic skin

**Fig. 7:**
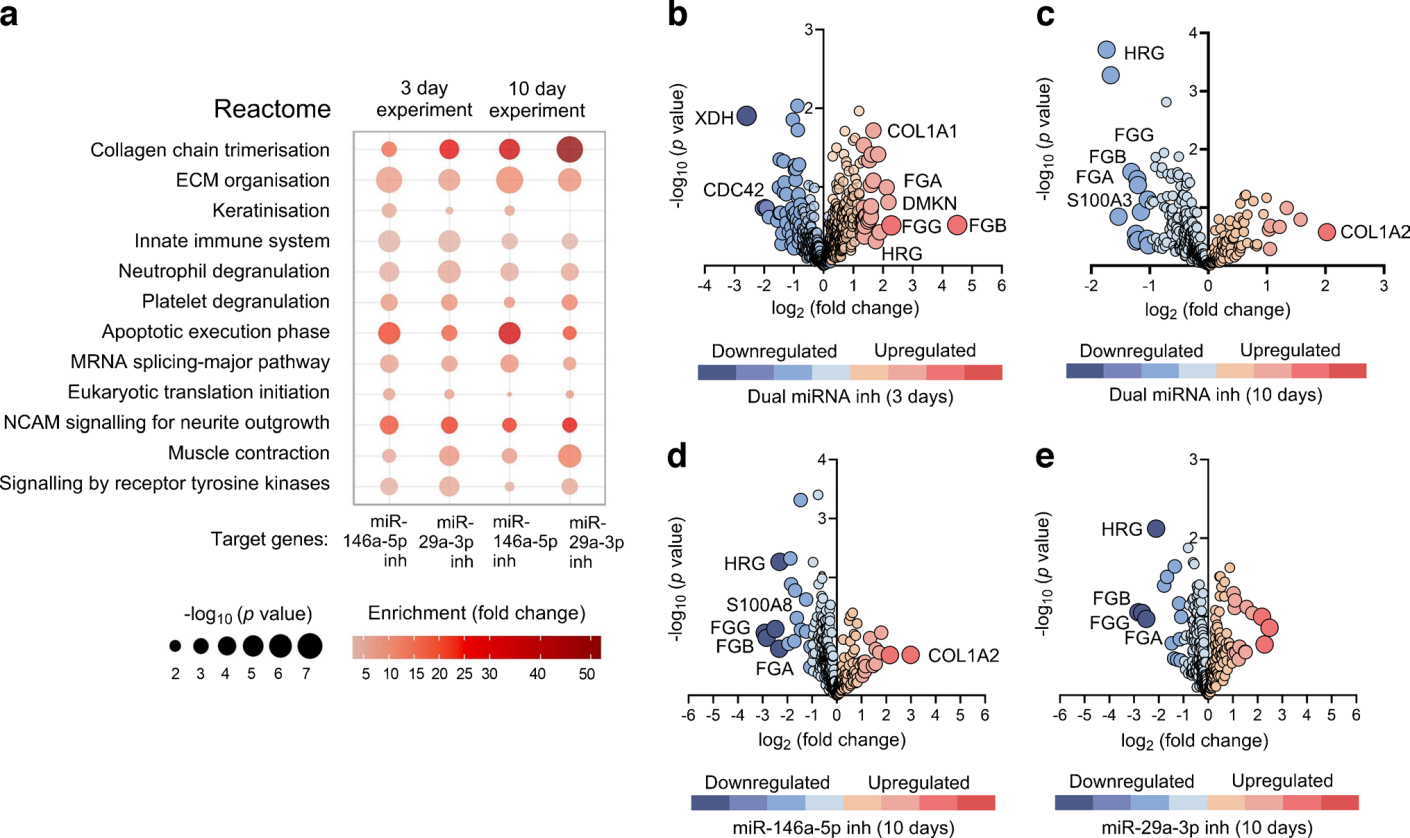
LC-MS/MS-based proteomics profiling of protein regulation in diabetic mouse skin. (**a**) Reactome pathway categories showing relative enrichment for genes targeted by individual miR-146a-5p or miR-29a-3p inhibitors, 3 and 10 days post wounding, in diabetic skin. Pathways are ranked for biological relevance based on the lowest *p* value of their components and fold change level of representation (ESM Table 6). (**b**–**e**) Volcano plots showing the distribution of proteins most relevant for wound healing according to *p* value and fold change (ESM Table 5), indicating a level of downregulation with blue and upregulation with red, with a colour-coded degree of difference presented as FDR values following the Fisher test. Inh, inhibitor, NCAM, neural cell adhesion molecule

Proteins common to the proteomic datasets (ESM Table 5) were further tested for functional enrichment in the Protein ANalysis THrough Evolutionary Relationships (PANTHER) database (ESM Table 6) [24]. Reactome pathway analysis revealed that inhibiting individual miRNAs mainly affected tissue spatial reorganisation in later wound healing stages, with high enrichment in dermal remodelling and collagen assembly (Fig. 7a). Contrastingly, the miR-29a-5p inhibition was associated with low or absent enrichment in the keratinisation. miR-146a-5p inhibition preferentially regulated neutrophil/platelet degranulation and apoptotic pathways, impacting stress responses in the inflammatory wound environment (Fig. 7a).

Several proteins amplifying the wound inflammatory signalling, such as xanthine dehydrogenase/oxidase (XDH), cell division cycle 42 (CDC42) and collagen 1α (COL1A), were suppressed, while dermokine (DMKN), T cell surface antigen CD2 (SRBC)/metalloproteinase 9 (MMP9), histidine-rich glycoprotein (HRG) and fibrin monomers (fibrinogen α chain [FGA], fibrinogen γ chain [FGG], fibrinogen β chain [FGB]) were increased at early wound healing stages (day 3) (Fig. 7b). By day 10, HRG, fibrin monomers and S100 proteins decreased, promoting collagen fibre assembly through dual miRNA inhibition (Fig. 7c), as well as by individual miRNA inhibition (Fig. 7d, e).

## Discussion

To the best of our knowledge, this is the first report on the dual inhibition of miR-146a-5p and miR-29a-3p and the effects on diabetic wound healing. While there are inconsistent reports on miR-146a-5p expression patterns during the progression of wound healing, its role in inflammation-driven cytokine secretion and cell activation responses tremendously dictates the non-healing outcome [10, 25, 26]. Many studies agree upon miR-146a’s direct interaction with target mRNAs such as those encoding IRAK1, IL receptor-associated kinase 2 (IRAK2) and TNF receptor-associated factor 6 (TRAF6), members of the NF-κB signalling cascade, directly via antisense oligos targeting miR-146 or indirectly via cell-derived exosomes with miR-146a-5p cargo targeting the diabetes-related wound healing impairments [7, 27, 28].

Increased in the later phase of skin epithelial wound healing, miR-29a-3p is suppressed by TGF-β-mediated phosphorylation of anti-fibrotic downstream targets such as SMAD, contributing to scar-free healing [29]. Conversely, miR-29 can control an additional subset of profibrotic proteins, including metalloproteinases, laminins and integrins, independently of TGF-β1 levels [30]. Furthermore, miR-29 family members have been unambiguously reported in controlling cellular functions such as cell–cell adhesion in the hyperproliferative mouse epidermis [8], cell differentiation [31] or p85α/CDC42-mediated apoptosis [32, 33].

Proteome profiling of HaCaT cells transfected with LNA-spiked antisense miR-146a-5p, miR-29a-3p or Scr oligos suggests the combined effect improves diabetic wound healing. The synergy between antimiR-146a-5p and -29a-3p creates positive or negative feedback loops, modulating specific protein levels to enhance healing.

A striking finding with the dual treatment was the disappearance of key proteins controlling the cell proliferation and migratory activity, such as LM332, compared with the individual inhibitors. For example, inflammatory signals from the wound fluid environment stimulate the overproduction of laminin 5 in human keratinocytes [34]. Moreover, a decrease in keratinocyte laminin levels in response to miR-29a-3p inhibition was previously shown to be associated with keratinocyte hyper-motility/growth arrest [8]. In conjunction with this observation, several laminin-binding integrins were increased, which could be due to lower levels of their laminin-binding partners [35]. Interestingly, both laminins and their ligands showed the opposite expression levels in cells stimulated with TNF-α [36, 37].

Further validation of our findings was done in an animal model of diabetic wound healing. In vivo*,* studies demonstrated meaningful improvements in wound healing with a simple dosing regimen, with just two topical applications per day for 3 days of miR-146a-5p or -29a-3p inhibitors, and particularly with a combination of both. While we expected that the interdependent actions observed in vitro would preferentially activate the immediate miRNA-146 keratinocyte-specific responses to breaching the skin barrier [1, 38], the deep matrix of the wound displayed macrophage phenotypic switching prevailing towards anti-inflammatory, possibly the reason for the observed reduction in superoxide levels. Moreover, keratinocytes and macrophages have known roles in secreting growth factors such as vascular endothelial growth factor and TGF-β, which can stimulate endothelial cells to restore the vascular network at early stages of wound healing [39, 40].

As CDC42 is important for re-epithelialisation, and CDC42/MMP9 mediates degradation of ECM and cleans up the wound site [41], their potential synchronous regulation by the dual miRNA inhibition at day 3 suggests a favourable effect on wound healing progression. Interestingly, individual miR-146a-5p inhibition resulted in higher COL1A levels and decreased levels of fibrin monomers FGA, FGB and FGG [42], as opposed to the suppression of COL1A fibres with miR-29a-3p inhibition [9]. Dual miRNA inhibition also increased the levels of COL1A1 in mouse skin, along with DMKN. Increased keratinocyte DMKN levels are associated with active wound healing [43], whereas COL1A fibrogenesis in fibroblasts aids tissue remodelling [44]. Dual miRNA inhibition initially elevates HRG levels to deposit more fibrin monomers, laying the foundation for faster coagulation response and denser vasculature earlier in wound healing. Moreover, the observed downregulation of S100 proteins at the wound site by dual miRNA inhibition indicates a reintroduction of immune homeostasis during wound maturation (at day 10) [45]. In a wound milieu, both keratinocytes and fibroblasts have the ability to synthesise ECM proteins such as collagen IV, collagen VII, perlecan and laminins, except α3 chain-containing laminins, in a time-dependent dynamic fashion [46]. The two laminins, 311 and 332, are synthesised by keratinocytes, with laminin 332 exclusively interacting with collagens VII and XVII to anchor basal keratinocytes to the underlying dermis, anchoring fibrils and hemidesmosomes [47].

Two of the chains, α3 and γ2, are post-translationally processed in fibroblasts, shaping the ECM architecture. This may explain why we observe transcriptional regulation by keratinocytes, but no laminin was detected in mouse biopsies [47, 48].

Altogether, these events enable the final stages of wound healing: fibroblasts deposit the foundation for the new connective tissue [49], and keratinocytes migrate over formed granulation tissue to remodel the wound [9].

The present study has several strengths. The study collectively showed that dual supplementation of miRNA antisense oligos improves wound healing. We present changes in a proteome profile of HaCaT cells (LC-MS/MS), transcriptional regulation (reporter gene analyses) induced by modulating the expression of specific miRNA levels in combination, substantiated by a temporal change in the expression levels of specific proteins (immunofluorescence, western blots, healing kinetics, histology), and proteome-wide alterations (LC-MS/MS) related to keratinocytes, fibroblasts, endothelial cells and immune cell activation towards the resurfacing with new epithelium in an animal wound healing model.

The present study also has important limitations. We chose to focus on a full-length 10 day STZ model study as it allowed us to observe an acute and comprehensive effect of dual inhibition on the network of miRNAs, as the wounds undergo the transition through overlapping phases of wound healing: the inflammatory phase (peaking at day 3), and the proliferation and remodelling phase (estimated at day 10). It should be noted that wound remodelling on a cellular level continues beyond day 10. Our animal model could be improved by extending the experiment beyond 10 days, and the treatment duration beyond days 1 to 3. Moreover, it was a limitation of the model that the wound size at day 10 could not be precisely assessed due to scab remnants, although histological analysis did indicate positive effects of dual miRNA inhibition. Thus, while cellular, histological and proteomic results support improved wound healing, the in vivo data should be interpreted with caution and would benefit from further studies. Moreover, there is a consensus that more than one animal model should be studied for reliable results. We acknowledge that the STZ-induced diabetic mouse model may not fully replicate the chronic, metabolic challenges in human diabetic wounds. Incorporating the *db*/*db* model and human ex vivo studies for follow-up studies would address this limitation and provide a more comprehensive understanding of our approach in a therapeutic context [50].

Although it is speculative to propose a common mechanism of regulation of wound healing, the observed impact on wound healing is intriguing. It raises questions such as whether it affects immune cell recruitment or cytokine secretion within the wound bed in response to laminin modulation and the potential compensatory mechanisms that may be triggered by laminin downregulation.

The translational meaning of our findings would be strengthened with validation in human studies [16]. Chronic wounds, including venous, pressure and diabetic foot ulcers, share common abnormalities, depending on diabetes span, age and associated health problems [51, 52].

Aberrant expression of circulating miRNAs has been reported in individuals with microvascular complications; however, only a limited number of studies have systematically investigated circulating miRNA signatures associated with diabetic complications [53]. As foot complications are a major problem in people with longstanding diabetes, miRNAs have a possible dual role as biomarkers and therapeutic targets to counteract the management of chronic non-healing wounds, possibly aided by an optimised delivery system, a concept proposed in several studies [54–57].

To gain a comprehensive understanding of multiple miRNA roles in wound repair, future studies should integrate single-cell studies, multi-cellular models or co-culture systems to better map the cellular crosstalk in dynamic miRNA regulation during wound healing.

As a final remark, the synergistic mechanisms underlying the wound healing response to dual miRNA inhibition remain to be completely elucidated. Our findings provide proof of concept that by modulating the faulty expression of specific miRNAs in diabetic skin, it is possible to counteract the adverse outcome of wound healing in diabetes.

## Supplementary Information

Below is the link to the electronic supplementary material.

ESM (PDF 2055 KB)

ESM Tables (XLSX 3.52 MB)

## Data Availability

The mass spectrometry proteomics data have been deposited to the ProteomeXchange Consortium via the PRIDE [58] partner repository with the dataset identifier PXD060087.

## Abbreviations

CDC42: Cell division cycle 42
COL1A: Collagen 1α
DHE: Dihydroethidium
DMKN: Dermokine
ECM: Extracellular matrix
FDR: False discovery rate
FGA: Fibrinogen α chain
FGB: Fibrinogen β chain
FGG: Fibrinogen γ chain
GO:CC: Gene Ontology Cellular Component
HRG: Histidine-rich glycoprotein
IRAK1: IL receptor-associated kinase 1
ITGA6: Integrin α6
ITGB1: Integrin β1
ITGB4: Integrin β4
LAD1: Ladinin 1
LAMA3: Laminin α3 chain
LAMB3: Laminin β3 chain
LAMC2: Laminin γ2 chain
LFQ: Label-free quantification
LM332: Laminin 5 complex
LNA: Locked nucleic acid
MMP9: Metalloproteinase 9
PCA: Principal component analysis
ROS: Reactive oxygen species
RT-qPCR: Reverse transcription with quantitative PCR
Scr: Scramble
STZ: Streptozocin
TNC: Tenascin C
XDH: Xanthine dehydrogenase/oxidase
