# Supplementary material for: Improved wound healing by dual inhibition of miR-146a-5p and miR-29a-3p supports a network action of dysregulated miRNAs in diabetic skin
Source: Diabetologia. 2025 Sep 3;69(1):214–29. doi: 10.1007/s00125-025-06522-3 (PMC12685990; doi:10.1007/s00125-025-06522-3)
Supplement: Supplementary file 1 — ESM (PDF 2055 KB) [file 125_2025_6522_MOESM1_ESM.pdf]

Improved wound healing by dual inhibition of miR-146a-5p and miR-29a-3p supports a network action of dysregulated miRNAs in diabetic skin

Marija Petkovic[1,2,3,4], Ermelindo C. Leal[3,4], Anja E. Sørensen[2], Per T. Jørgensen[5], Jesper T. Wengel[5,6], Rosa R. Jersie-Christensen[2], Jesper T. Troelsen[2], Eugenia Carvalho[3,4]*, and Louise T. Dalgaard[2*]

[1]Steno Diabetes Center Copenhagen, Herlev Hospital, Herlev, Denmark

[2]Department of Science and Environment, Roskilde University, Roskilde, Denmark

[3]CNC-UC- Centre for Neuroscience and Cell Biology, CIBB- Centre for Innovative Biomedicine and Biotechnology, University of Coimbra, Coimbra, Portugal

[4]Institute for Interdisciplinary Research, University of Coimbra, Coimbra, Portugal

[5]Department of Physics, Chemistry and Pharmacy, University of Southern Denmark, Odense, Denmark

[6]Faculty of Engineering and Science, Aalborg University, Aalborg, Denmark

*Shared corresponding authorship: Eugenia Carvalho, Center for Neuroscience and Cell Biology, University of Coimbra, Polo I, Rua Larga 1, 3004-504 Coimbra, Portugal; Phone +351 239 820 190; email: ecarvalh@cnc.uc.pt and Louise T Dalgaard, Department of Science and Environment, Universitetsvej 1, Roskilde University, 4000 Roskilde, Denmark. Phone: +45 46 74 2713. E-mail: ltd@ruc.dk

Electronic supplementary materials (ESM)

Contents:

ESM Methods

ESM Table 1 List of oligonucleotides

ESM Table 2 miRDB results

ESM Table 3 MaxQuant_ HaCaT_oligos (Excel file)

ESM Table 4 MaxQuant_ HaCaT_TNF-α (Excel file)

ESM Table 5 MaxQuant_ C57BL6 (Excel file)

ESM Table 6 Reactome_ C57BL6 (Excel file)

ESM Fig. 1 IRAK1 and p85 protein expression

ESM Fig. 2 HaCaT proteome after miRNA inh and TNF-α stimulation

ESM Fig. 3 HaCaT proteome after miRNA inh and TNF-α stimulation (extension)

ESM Fig. 4 Haematoxylin-Eosin staining of wound sections

ESM Fig. 5 Macrophages_Immunofluorescence_complete figure

ESM Methods

HaCaT cell model

Immortalised keratinocytes (HaCaT cells) (RRID: CVCL_0038; a gift from Håvard Jenssen) were maintained in Dulbecco's modified Eagle medium (DMEM) with 25 mM glucose, containing glutamax, supplemented with 10% FBS, and 1% Penicillin/Streptomycin. Cells were tested for mycoplasma by staining DNA and observation for cytoplasmic DNA stain. For experiments, cells were seeded at 45,000 cells per well and allowed to adhere for 24 hours) [1]. Following media change, cells were stimulated with TNF-α (10ng/ml), n=6 wells/group; or transfections were made (n=4 wells/group) with 25 pmol LNA (locked nucleic acid)-spiked fully phosphorothioate modified antisense miR-146a-5p (5'-TGgaauucAgTuCucA-3'), miR-29a-3p (5'-AuuTcaGauGguGcuA-3') oligos (LNA bases are written in capital letters while 2′-O-methyl RNA bases in small letters) and a scramble (Scr) control oligo (5'-CaaTagGguCaaGauT-3') individually or in combination (1.25 pmol of each inhibitor), and Lipofectamine 2000 (ThermoFisher Scientific) according to the manufacturer's instructions. After 24 hours, the medium was changed to remove non-adherent cells and cell lysates were harvested for proteomic analysis.

Animal type 1 diabetes model of wound healing impairments

Male C57BL6 mice (5 or 6 animals per group (see below), 25-30g, Charles River Inc., Barcelona, Spain) were maintained on a 12-h light/12-h dark cycle, at 22-24°C with access to water and chow food ad libitum. Animal protocols followed the European Community law for Experimental Animal studies (86/609/CEE and 2007/526/CE) and were approved by the Institutional and Ethical Board at the University of Coimbra and by the Governmental (Directorate-General for Food and Veterinary of the Portuguese Ministry of Agriculture) Research Ethical Boards. Diabetes was induced by 5 low-dose intraperitoneal injections of streptozotocin (50 mg/kg) (Sigma-Aldrich). Blood glucose was measured one week after the last injection. Mice that met inclusion criteria of having the glucose levels >14 mmol/l were considered diabetic, as previously described [1, 2]. Six weeks after diabetes induction, two full-thickness bilateral dorsal wounds per mouse were made with a 6 mm punch biopsy tool (Kai Europe Gmbh, Germany), as described previously [3]. Mice were randomly allocated to treatment groups.

a) MicroRNA levels in normoglycaemic vs diabetic mice skin

Type 1 diabetes was induced in male C57BL6 mice (6 animals per group, 25-30g, Charles River Inc., Barcelona, Spain). Normoglycaemic (non-diabetic) mice (6 animals per group) were used as controls. Animals were pre-treated with in-house analgetic Buprenorphine (0.1 mg/kg) and anaesthetised by inhaling 2.5% isoflurane combined with oxygen (0.5 l/min) isoflurane before two circular 6-mm full-thickness excisional wounds extending through the panniculus carnosus on the dorsum 1cm to either side of the midline and halfway between the shoulders and the pelvis were introduced. Three days after the wounding (corresponding to the normal inflammatory phase of wound healing) or 10 days after the wounding (corresponding to granulation/proliferation and matrix remodelling phase), respectively, animals were euthanised by Ketamine/Xylazine (Vetoquinol, Bayer, Germany) (300/30 mg/kg, intraperitoneally) and cervical dislocation, for biopsy collection. Harvested skin tissues were cryopreserved at -80°C for RNA extraction and detection of miRNA expression levels. The number of mice per experimental group was determined based on the following power analysis: Equal sample size and variability (35% relative to mean) among the groups, at $p<0.05$, with 80% power and 50% expected difference in miRNA expression between groups, was found to be n=6/group. Our previous experiments on streptozotocin-induced diabetes indicate that nearly 100% of the treated mice develop diabetes with very low mortality (2-5%).

b) Topical treatments with miRNA inhibitors

Diabetes induction and wound healing procedure was performed in the same manner as described in the previous section.

The dose regimen, including the schedule of topical application twice daily, duration of treatment up to day 3 post wounding and the amount of miR-146a-5p inhibitor or miR-29a-3p inhibitor individually (2.5 nmol) or as a dual treatment (1.25 nmol each inhibitor) or with a scramble miR-inhibitor as negative controls to be for delivery in vivo in a small volume (5µl), was decided according to published literature related to similar studies on miRNA antagonists and other topical delivery systems in wound healing experiments [4–9].

The individual performing the measurements was not blinded to the treatment allocation because the animals were handled during post-surgery recovery (e.g. analgesia administration) by the same person but was blinded to the treatment allocation during assessments of wound area obtained from acetate tracing. This ensured that the

measurements and analyses were conducted objectively, without knowledge of the experimental group assignments.

The study design included two groups of animals. Three days experimental group (5 animals per group): dual treatment (1.25 nmol each inhibitor) and a scramble miR-inhibition as negative controls. The ten-day experimental group (5 animals per group) involved: miR-146a-5p inhibitor or miR-29a-3p inhibitor individually (2.5 nmol) or as a dual treatment (1.25 nmol each inhibitor) and a scramble miR-inhibitors treated wounds. The number of mice per experimental group was determined based on the following power analysis: Equal sample size and variability (17.5 % relative to mean) among the groups, at $p<0.05$, with 80% power and 30% expected difference in wound size between groups, was found to be n=5/group.

Harvested skin tissues were cryopreserved at -80°C for further analyses or embedded in optimal cutting temperature (OCT) medium (VWR) and stored at -80°C or embedded into paraffine at room temperature.

RNA extraction and RT-qPCR

Skin tissue (50-100mg) was homogenised with 1ml TRI Reagent (Sigma Aldrich, St. Louis, Missouri, USA) with a Tissue Lyser II (Qiagen, Germantown, Maryland, USA) followed by purification as per manufacturers' instructions. The RNA pellet was dissolved in DEPC water (50µl). RNA concentration and purity were assessed using the Nanodrop ND-1000 spectrophotometer (ThermoFisher Scientific, Waltham, Massachusetts, USA) and stored at −80°C.

MiRNAs were detected using reverse-transcription quantitative PCR (RT-Q-PCR) as previously described [10]. Briefly, cDNA was primed using specific miRNA RT primers and synthesised using Superscript III reverse transcriptase (ThermoFisher Scientific). Oligonucleotides used for reverse transcription and Q-PCR are shown in ESM Table 1. MiRNA levels were quantified using Quantitect Sybr 2x Master Mix (Qiagen, Hilden, Germany) in 10 µl reactions using the MX3005 qPCR system (Agilent, Santa Clara, California, USA). Transcripts were quantified using standard curve quantification, and diluted skin cDNA served as input to generate the standard curve. The geometric mean of transcription factor TFIIB and U6 levels were used to normalise for variation in the input template. The geometric mean of these two transcripts normalised to the control baseline was unaltered between treatment groups.

Data were analysed by two-way ANOVA with Tukey's post hoc correction; comparing the difference of miR-146a-5p and -29a-3p expression levels before and after wounding between normoglycemic and diabetic group; or difference between miR-146a-5p and -29a-3p expression levels in diabetic group pre- and 3-and 10- days post wounding).

Proteomic analyses

LC-MS/MS acquisition conditions

Whole-cell lysates (HaCaT keratinocytes; n=4 wells/group for miR inhibition; n=6 wells/group for TNF-α stimulation experiment) (100µl); or skin biopsies (C57BL6 mice; n=5 animals/group) (20µg) homogenised with Tissue Lyser II (Qiagen, Germantown, Maryland, USA) were prepared using an ice-cold lysis buffer containing 6M Guanidinium hydrochloride (GuHCl), 5 mM Tris (2-carboxyethyl) phosphine (TCEP), 10 mM chloroacetamide (CAA), 100 mM Tris–HCl pH 8.5 [11].

Protein concentration was measured with Pierce™ BCA Protein Assay Kit (23225, Thermo Fischer, US), and samples were digested with trypsin protease, MS Grade (Pierce, Thermo Scientific). We performed a bottom-up (also known as shotgun), label-free quantification (LFQ) proteomics (no protein labelling was applied to the proteins analysed in this study). Peptides (200µg) were cleaned up using silica columns (Sep-Pak C18, Waters, USA) and concentrated using speed-vac.

Generated label-free peptides (5µg proteins) were subjected to high–resolution Mass Spectrometry LC-MS/MS. All LCMS/MS specimens were run in three consecutive runs as a technical replicate, using the following setup:

Peptides were trapped on a C18 column (5 µm, 5mm, 0.3mm) and separated on a 15 cm fused silica column (75 µm inner diameter) pulled and packed in-house with 1.9 µm C18 beads (Reprosil-AQ Pur) on an Ultimate 3000 system connected to a LTQ Velos Orbitrap (Thermo Scientific, San Jose, US). Peptides were separated with a 110 min gradient with increasing buffer B (90% ACN and 0.1% formic acid), going from 5% to 30% in 70 min, 30 to 50% in 15 min, 50 to 95% in 20 min followed by a 5 min wash and re-equilibrating step. All steps were performed at 250 nL/min flow rate and 40°C.

The LTQ Velos Orbitrap was operated in data-dependent top 15 mode. Full scan mass spectra were recorded in the orbitrap at a resolution of 60,000 at m/z 200 over the m/z range 375–1600 with a target value of 1×10^6 and a maximum injection time of 500 ms. CID-

generated product ions were recorded in the ion trap with a maximum ion injection time set to 100 ms and a target value set to 1×10^4.

The spray voltage was set to 2.2 kV, the S-lens RF level at 50, and the heated capillary at 300°C. Normalised collision energy was set at 35 and the isolation window was 2 m/z.

Proteomic data handling and statistical analysis

Raw data were processed with MaxQuant v1.6.12.0 with default settings including LFQ and searched against UniProtKB: H. sapiens or M. musculus reference proteome. LFQ values were used for data interpretation and initial statistical analysis employing Perseus software 1.6.15.0 to identify the differentially expressed proteins in cell /biopsy lysates.

Two skin biopsies had failed reactions for proteomics: One sample for miR-146a-5p inh. and one for scramble treatment, giving 18 samples in total, with four or five samples per group.

After data filtering, to normalise the distribution, we performed $\log_2$ transformation of the intensity data, which is the default setting. A pool of “NaN” (Non-Assigned Number) values, corresponding to expression signal values below the threshold, was resolved by filtering for valid values using embedded settings of filtering rows based on valid values such that every row contains at least 3 valid values per group to ensure data analysis of robustly detected proteins. For principal component analysis (PCA) the algorithm required subsetting the data set to contain only proteins expressed in all samples (all values valid).

Gene Ontology (GO) enrichment analysis for GO terms overrepresentation of the proteins that are significantly changing between the treatments was done Fisher exact test in Perseus [12] while pathway analyses were made using Panther. Db [13].

Statistical analyses of differences between the groups were performed using a t-test with corrections for multiple tests/ one-way or two-way ANOVA followed by followed by Tukey's post hoc correction or Permutation-based FDR correction for multiple hypotheses testing in Perseus 1.6.15.0. The p values lower than 0.05 were considered statistically significant.

Reporter-gene analysis

The LAMC2 promoter and enhancer (luciferase-coupled) constructs were described previously [4]. For reporter-gene analysis, HaCaT cells (n=6 wells/group) were seeded in 48 well plates (45,000 cells per well) and allowed to adhere for 24hrs. Each transfection contained 450ng LAMC2/luciferase construct, with or without miR inhibitors. After 24 hours, cells were harvested and lysed, and luciferase activities were determined using the Dual Light® system (Tropix; Perkin Elmer) using a GloMax96 instrument (Promega) according to

the manufacturer's instructions. Protein concentration was measured by the BCA method. Transfections for LAMC2 promoter activities were performed in sextuplicate for each promoter, and promoter activity was normalised to the protein content in the cell extracts. The results are presented as mean±SD of three independent experiments, two-way ANOVA, with Tukey post hoc correction, comparing the promoter activity between the non-stimulated and TNF-α-stimulated cells; or miR-146a-5p/-29a-3p inhibition-driven promoter activity relative to Scr inhibition promoter activity.

Immunofluorescence

The M1/M2 macrophages ratio, and the rate of neovascularisation, were measured with immunofluorescent staining. Rabbit polyclonal anti-CD68 (ab125212, Abcam, Cambridge, UK), rat monoclonal anti- TNF-α (MCA1488, AbD Serotec, Algés, Portugal), rat monoclonal anti-CD206 (MR5D3) (sc-58987, Santa Cruz, Santa Cruz, USA) and rat monoclonal anti-CD31 (PECAM-1) (CBL1337, Merck Millipore, Darmstadt, Germany) antibodies were diluted 1:100 in 2% BSA (Bovine Serum Albumin) and 1% normal goat serum (Novex, Life Technologies). OCT Frozen sections (8µm thickness) were permeabilised, fixated with ice-cold acetone (VWR) and blocked with 5% BSA/normal goat serum. Positive staining was indicated as intense red (TNF-α, CD31, CD206) or bright green (CD68) positive-coloured cells or vessel branches after incubation with anti-rat (conjugated to Alexa Fluor 568, Invitrogen) and anti-rabbit (Alexa Fluor 468 conjugated, Invitrogen) secondary antibodies, diluted 1:500 in 5% BSA and 5% normal goat serum. The nuclei were stained with DAPI (D9542 Sigma, St. Louis, MO). Fluorescent images were obtained with a confocal microscope (Zeiss LSM 510 Meta), using Zen blue 2.1 SP3 software (Carl Zeiss) with 200 times and 400 times magnifications. The number of macrophages cell abundance (3 selected areas for a cell count of 6 pictures per sample) or branching vessels (5 images for each sample) expressing fluorescent signal were quantified as the average number per field with ImageJ2 software and normalised to the average number of cells/vessels in biopsies at the baseline. The areas for cell/vessel counting were pre-specified before the quantification so that 3 regions of interest (ROI) created in ImageJ2 (same size across all images quantified) would cover regions of the image where the section is located (aiming for epidermis/dermis in the wound bed) in a random manner, avoiding areas with artefacts or poor signal quality. To minimize potential bias, the assessor was blinded to the treatment labels on microscopy slides during the analysis slides until the analysis was completed. Data

are presented as mean ± SD, one-way ANOVA, followed by Tukey's post hoc correction, comparing the miR-146a-5p/-29a-3p inhibition effect on M1, M2 and M1/M2 ratios, or number of blood vessels relative to the Scr inhibition effect; or miR-146a-5p/-29a-3p inhibition/ Scr inhibition macrophage numbers/vessels count changes relative to day 0 diabetic skin baseline values.

Fluorescence analysis for ROS detection

Dihydroethidium is widely used to detect superoxide anion O_2^- formation [16, 17], the detection of which results in a shift from blue to red fluorescence. DHE staining was performed on (30µm) OCT embedded frozen sections following manufacturers' instructions (ThermoFisher Scientific). Briefly, DHE (10uM) was applied followed by incubation for 30 minutes at 37ºC, in a humidified dark chamber, and then fixation with paraformaldehyde 4% for 5 min at RT. Slides were counterstained with DAPI for nuclei labelling (Sigma, USA). Fluorescent images were acquired with a confocal microscope (Zeiss LSM 510 Meta) at 200 times magnification (6 pictures/5 random square fields per picture for each sample). The areas for fluorescent signal were pre-specified before the quantification so that 5 regions of interest (ROI) created in ImageJ2 (same size across all images quantified) would cover regions of the image where the section is located (aiming for epidermis/dermis in the wound bed) in a random manner, avoiding areas with artefacts or poor signal quality. To minimize potential bias, the assessor was blinded to the treatment labels on microscopy slides during the analysis slides until the analysis was completed. Densitometric analysis of the DHE signal was measured as the integrated density grey mean values quantified in ImageJ2 software. Data were normalised to the average integrated density value in biopsies at the baseline. Data are presented as mean ± SD, one-way ANOVA, followed Tukey's correction for differences in DHE signal of miR-146a-5p/-29a-3p inhibition relative to 29a-3p inhibition/Scr relative to DHE signal corresponding to day 0 baseline values.

Histological analyses

Formalin-fixed and paraffin-embedded skin sections (3µm) were stained using Masson Goldner Trichrome (Carl Roth, Germany), according to manufacturers' protocol, selectively staining the connective tissue green, the nuclei brown and the cytoplasm red. Sections were also stained with haematoxylin (Harris haematoxylin (Sigma-Aldrich, Germany)) and eosin (Sigma, Portugal). Images were acquired using a Zeiss Axiovert Imager wide-field microscope. Collagen distribution in the dermis was quantified as the mean integrated

density for the green channel after image deconvolution. Ten distinct sites per section were measured and averaged to determine the relative dermal collagen density, using 200 times magnification and ImageJ2 software (NIH, USA). For image analyses, the identities of samples were blinded to the observer. Three sections did not meet the imaging quality requirements (the signal remains within the linear range for quantification) due to high saturation with MT stains, thus were excluded from analysis: one section from each of the Scr inh, miR-146a-5p inh and the dual miRNA treatments, giving 17 samples in total, with four samples per group. Data are presented as mean ± SD, one-way ANOVA, followed Tukey's correction for differences in mean integrated density for the green channel signal of miR-146a-5p/-29a-3p inhibition relative to Scr inhibition values.

Western blots

Skin tissue (n=5 animals) was homogenised in RIPA lysis buffer. Protein concentration was determined using the BCA kit. 30μg of total protein was resolved on 7.5% SDS-PAGE gel and then transferred to a PVDF membrane. The membranes were blocked with 5% BSA Fraction V (A4503, ThermoFisher Scientific) and probed with the following antibodies: Primary rabbit anti-IRAK-1 (ADI-905-709-100, Enzo Life Sciences, dilution 1:500), primary rabbit anti-p85 polyclonal (06-195, Millipore, diluted 1:1000), and secondary Goat anti-Rabbit IgG-(H+L)-HRP (314621, ThermoFisher, diluted 1:5000) and Goat anti-Goat IgG-(H+L)-HRP (31402, ThermoFisher, diluted 1:5000). Membranes were revealed with ESCL substrate (Pierce, ThermoScientific) and visualised with a ChemiDoc Touch Gel Imaging System (Bio-Rad laboratories). The data were normalised to goat anti-β-Actin (sc-1616, Santa Cruz Biotechnology, diluted 1:1000), and to average value in biopsies at the baseline. The densiometric analyses were performed in the Image Lab Software 6 (Bio-Rad Laboratories, Amadora, Portugal). Data are presented as mean ± SD, one-way ANOVA of three independent experiments (n=5 animals), followed Tukey's correction for differences in mean IRAK1 or p85 protein expression after the miR-146a-5p/-29a-3p inhibition relative to Scr inhibition values.

References

1. Moura LIF, Dias AMA, Leal EC, Carvalho L, Sousa HC De, Carvalho E (2014) Acta Biomaterialia Chitosan-based dressings loaded with neurotensin- an efficient strategy to improve early diabetic wound healing. Acta Biomater 10(2):843–857. https://doi.org/10.1016/j.actbio.2013.09.040

2. Leal EC, Carvalho E, Tellechea A, et al (2015) Substance P Promotes Wound Healing in Diabetes by Modulating Inflammation and Macrophage Phenotype. Am J Pathol 185(6):1638–1648. https://doi.org/10.1016/j.ajpath.2015.02.011

3. Moura J, Sørensen A, Leal EC, et al (2019) microRNA-155 inhibition restores Fibroblast Growth Factor 7 expression in diabetic skin and decreases wound inflammation. Sci Rep 9(1):5836. https://doi.org/10.1038/s41598-019-42309-4

4. Van Solingen C, Araldi E, Chamorro-Jorganes A, et al (2014) Improved repair of dermal wounds in mice lacking microRNA-155. J Cell Mol Med 18(6):1104–1112. https://doi.org/10.1111/jcmm.12255

5. Worm J, Stenvang J, Petri A, et al (2009) Silencing of microRNA-155 in mice during acute inflammatory response leads to derepression of c/ebp Beta and down-regulation of G-CSF. Nucleic Acids Res 37(17):5784–5792. https://doi.org/10.1093/nar/gkp577

6. Yi R, Poy MN, Stoffel M, Fuchs E (2008) A skin microRNA promotes differentiation by repressing "stemness." Nature 452(7184):225–229. https://doi.org/10.1038/nature06642

7. Ma X, Kumar M, Choudhury SN, et al (2011) Loss of the *miR-21* allele elevates the expression of its target genes and reduces tumorigenesis. Proceedings of the National Academy of Sciences 108(25):10144–10149. https://doi.org/10.1073/pnas.1103735108

8. Pastar I, Khan AA, Stojadinovic O, et al (2012) Induction of specific microRNAs inhibits cutaneous wound healing. Journal of Biological Chemistry 287(35):29324–29335. https://doi.org/10.1074/jbc.M112.382135

9. Yang LL, Liu JQ, Bai XZ, et al (2014) Acute downregulation of miR-155 at wound sites leads to a reduced fibrosis through attenuating inflammatory response. Biochem Biophys Res Commun 453(1):153–159. https://doi.org/https://doi.org/10.1016/j.bbrc.2014.09.077

10. Sørensen AE, Wissing ML, Englund ALM, Dalgaard LT (2016) MicroRNA species in follicular fluid associating with polycystic ovary syndrome and related intermediary phenotypes. Journal of Clinical Endocrinology and Metabolism. https://doi.org/10.1210/jc.2015-3588

11. Jersie-Christensen RR, Sultan A, Olsen J V. (2016) Simple and Reproducible Sample Preparation for Single-Shot Phosphoproteomics with High Sensitivity.

In: Methods in Molecular Biology, von Stechow L. (eds). Springer, New York, NY, pp 251–260

12. Jersie-Christensen RR, Lanigan LT, Lyon D, et al (2018) Quantitative metaproteomics of medieval dental calculus reveals individual oral health status. Nat Commun 9(1):4744. https://doi.org/10.1038/s41467-018-07148-3

13. Thomas PD, Ebert D, Muruganujan A, Mushayahama T, Albou L, Mi H (2022) PANTHER: Making genome-scale phylogenetics accessible to all. Protein Science 31(1):8–22. https://doi.org/10.1002/pro.4218

14. Boyd M, Coskun M, Lilje B, et al (2014) Identification of TNF-α-responsive promoters and enhancers in the intestinal epithelial cell model caco-2. DNA Research 21(6):569–583. https://doi.org/10.1093/dnares/dsu022

15. Coskun M, Boyd M, Olsen J, Troelsen JT (2010) Control of intestinal promoter activity of the cellular migratory regulator gene ELMO3 by CDX2 and SP1. J Cell Biochem 109(6):1118–1128. https://doi.org/10.1002/jcb.22490

16. Krauss S, Zhang C-Y, Scorrano L, et al (2003) Superoxide-mediated activation of uncoupling protein 2 causes pancreatic β cell dysfunction. Journal of Clinical Investigation 112(12):1831–1842. https://doi.org/10.1172/JCI200319774

17. Griendling KK, Touyz RM, Zweier JL, et al (2016) Measurement of Reactive Oxygen Species, Reactive Nitrogen Species, and Redox-Dependent Signalling in the Cardiovascular System. Circ Res 119(5): e39–e75. https://doi.org/10.1161/RES.0000000000000110

ESM Table 1

URP universal	Reverse	5’ TGG TGT CGT GGA GTC G
miR-29a-3p looped	Reverse	5’ TCAACTGGTGTCGTGGAGTCGGCAATTCAGTTGAGTAACCGAT
miR-29a-3p	Forward	AC ACT CCA GCT GGG TAG CAC CAT CTG AAA T
miR-146a-5p looped	Reverse	CTCAACTGGTGTCGTGGAGTCGGCAATTCAGTTGAGAACCCATC
miR-146a-5p	Forward	5’ ACACTCCAGCTGGGTGAGAACTGAATTC
TFIIB	Reverse	5’ TGTGTAGCTGCCATCTGCACTT
	Forward	5’ GTTCTGCTCCAACCTTTGCCT
U6	Reverse	5’ AACGCTTCACGAATTTGCGT
	Forward	5’ CTCGCTTCGGCAGCACA

ESM Table 1 - Oligonucleotide sequences used for RT-qPCR.

ESM Fig. 1

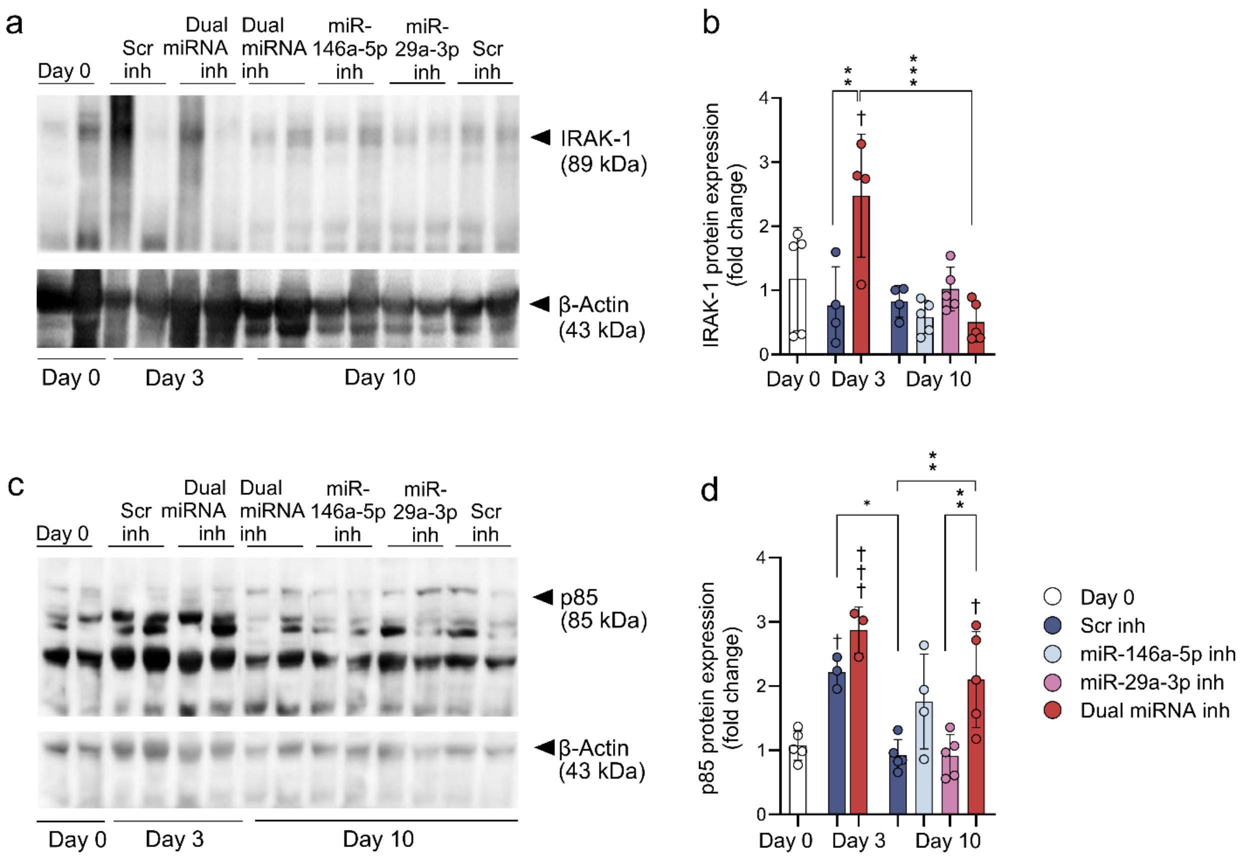

ESM Fig. 1 Relative IRAK-1 and p85 protein expression in wound skin. The difference in levels of IRAK-1 and p85 protein expression between animal groups (n=5) treated topically with 2.5 nmol of Scr, miR-146a-5p or -29a-3p inhibitors separately or in combination was measured 3 and 10 days after wounding in wound tissue lysates (30μg of proteins). (a) Representative blot image of IRAK-1 (b) Quantification of IRAK-1 protein expression (c) representative blot image of p85 (d) p85 protein expression measured by western blots. Data were analysed using Image Lab 6.0 software and are presented as mean ± SD of three independent experiments, one-way ANOVA, followed by Tukey's correction. **p*<0.05, ***p*<0.01, ****p*<0.001 (referring to miR-146a-5p/-29a-3p inhibition relative to Scr); and †*p*<0.05, †††*p*<0.001 (miR-146a-5p/-29a-3p inhibition/ Scr relative to day 0 baseline values).

ESM Fig. 2

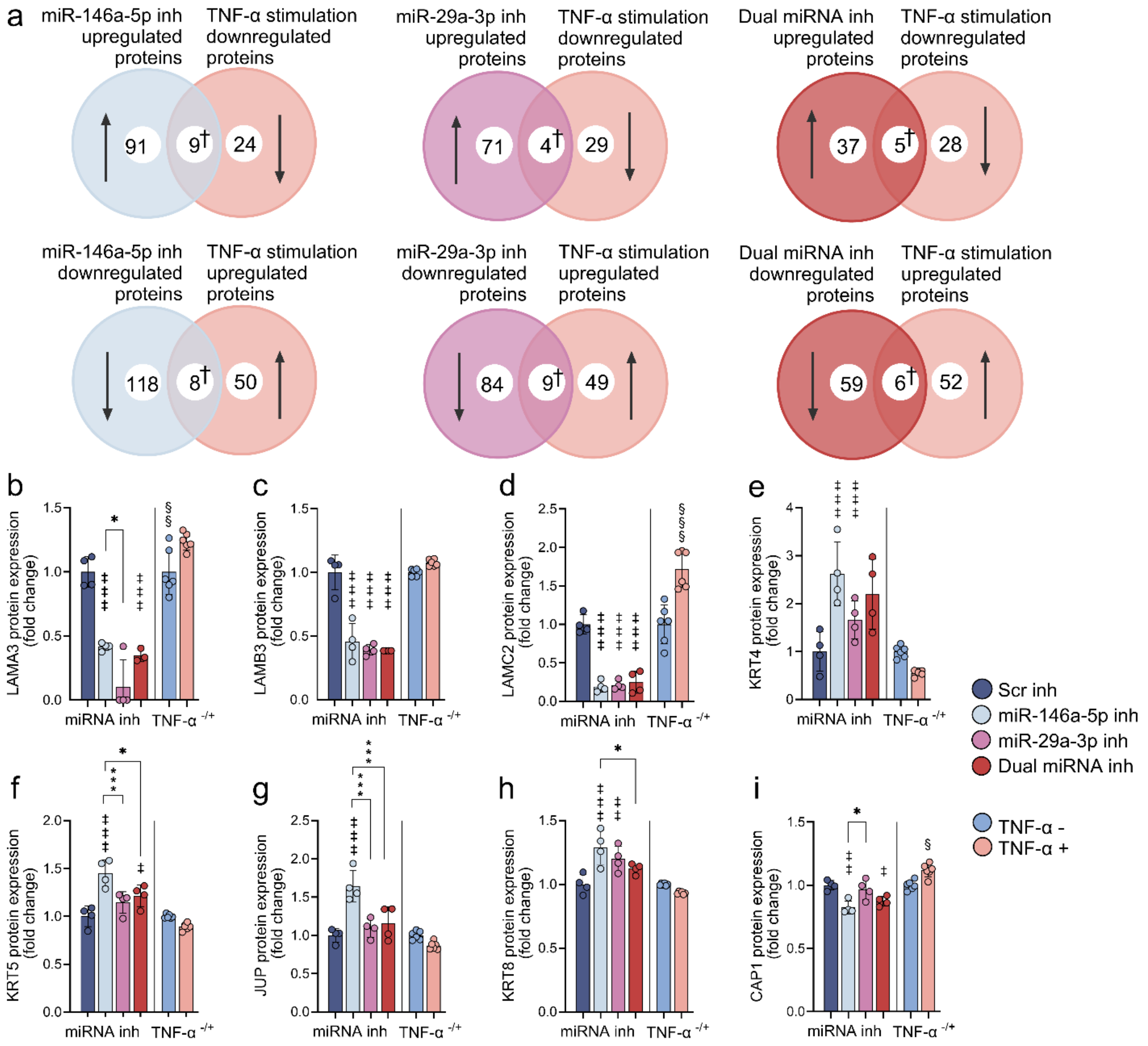

ESM Fig. 2 Protein profile of HaCaT cells after single or dual inhibition of miR-146-5p and -29a-3p or oppositely regulated by TNF-α signalling. (a) Venn diagrams illustrate the overlap of proteins significantly up- and down-regulated by miR-146a-5p and -29a-3p inhibition (n=4 wells/group) and proteins regulated in response to TNF-α stimulation (n=6 wells/group) (†significant regulation compared to non-treated HaCaT cells baseline values), (b)-(i) Expression levels of proteins changed by the treatments and significantly regulated by either individual or dual miR inhibitors actions or TNF-α. *p<0.05,**p<0.01,***p<0.001 (referring to miR-146a-5p/-29a-3p inhibition relative to Scr); §p<0.05, §§p<0.01 (comparing the effect of exposing HaCaT cells to the TNF-α), and ‡p<0.05, ‡‡p<0.01, ‡‡‡p<0.001 (miR-146a-5p/-29a-3p inhibition/ Scr relative to non-treated HaCaT cells baseline values). Abbreviations: LAMA3- Laminin subunit alpha-3; LAMB3- Laminin subunit beta-3; LAMC2- Laminin subunit gamma-2; KRT4- Keratin 4; KRT5-Keratin 5; KRT8- Keratin 8; JUP- Junction plakoglobin; CAP1- Adenylyl cyclase-associated protein 1.

ESM Fig. 3

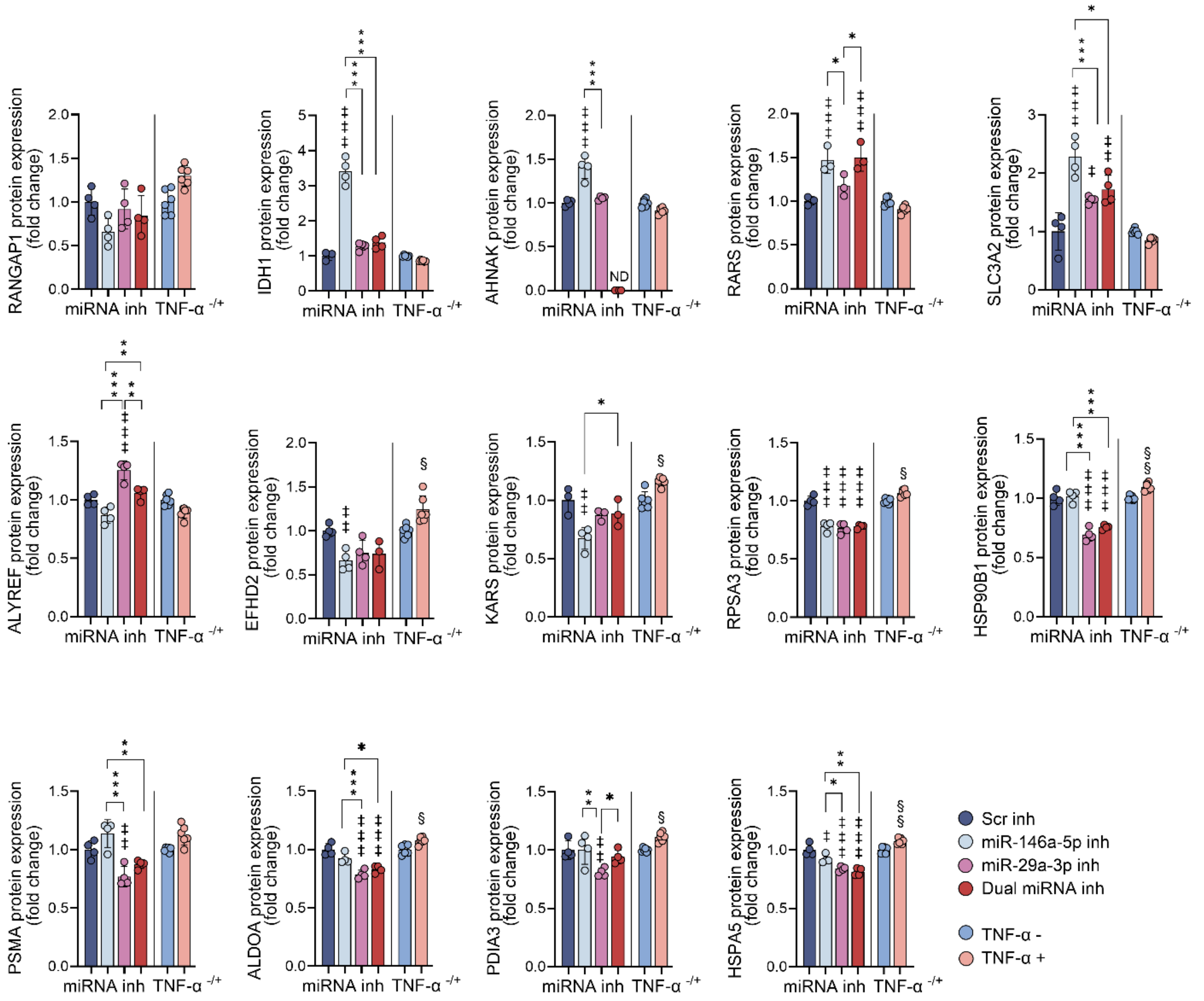

ESM Fig. 3 Proteins regulated by single or dual inhibition of miR-146 and -29a (n=4 wells/group), and oppositely regulated by TNF-α signalling in HaCaT cells (n=6 wells/group). *$p<0.05$, **$p<0.01$, ***$p<0.001$ referring to miR-146a-5p/-29a-3p inhibition relative to Scr; §$p<0.05$, §§$p<0.01$, comparing the effect of exposing HaCaT cells to the TNF-α, and ‡$p<0.05$, ‡‡$p<0.01$, ‡‡‡$p<0.001$ (miR-146a-5p/-29a-3p inhibition/ Scr relative to non-treated HaCaT cells baseline values). Abbreviations: RANGAP1- Ran GTPase-activating protein; IDH1- Isocitrate dehydrogenase [NADP]; AHNAK-Neuroblast differentiation protein; RARS- Arginine-tRNA ligase; SLC3A2-4F2 cell-surface antigen heavy chain; ALYREF-THO complex subunit 4; EFHD2- EF-hand domain-containing protein D2; KARS- Lysine-tRNA ligase; RPS3A- 40S ribosomal protein S3a; HSP90B1- Endoplasmin; PSMA6- Proteasome subunit alpha type-6; ALDOA- Fructose-bisphosphate aldolase A; PDIA3- Protein disulfide isomerase A3; HSPA5- 78 kDa glucose-regulated protein.

ESM Fig. 4

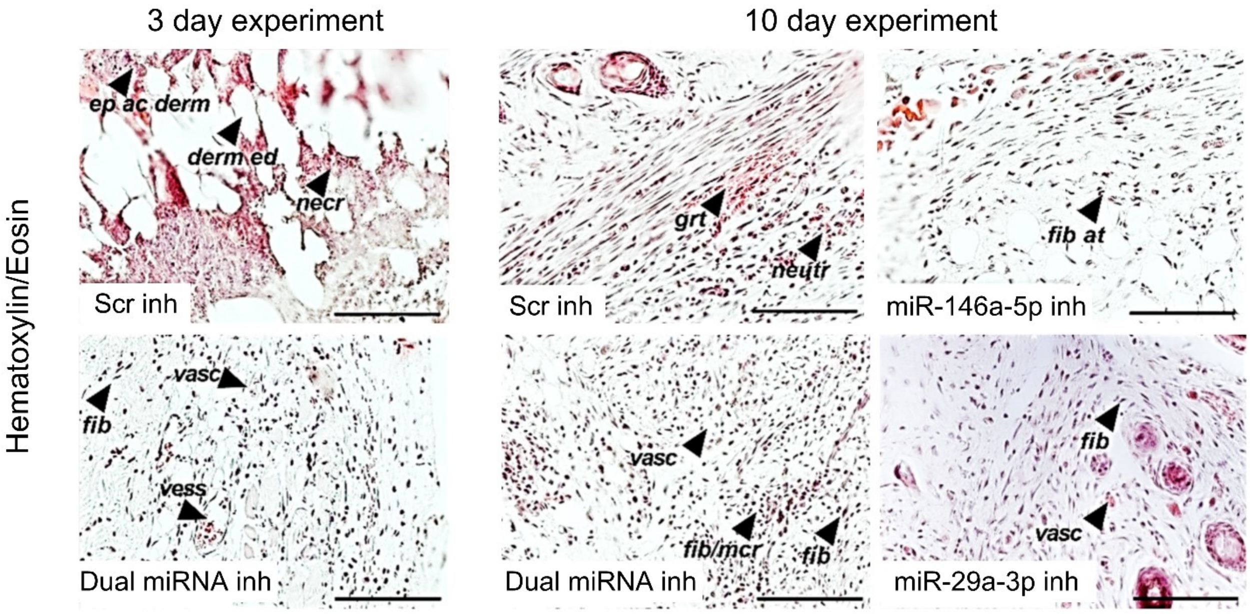

ESM Fig. 4 Improved inflammatory response and tissue regeneration after dermal treatments with single or combined miR-146a-5p and 29a-3p inhibitors. Representative microscopy images of Hematoxylin-Eosin (HE) staining of diabetic wound skin harvested 3 and 10 days after wounding following the treatment with Scr (negative control) or 2.5 nmol of miRs-146a-5p or -29a-3p inhibitors individually or in combination (1.25nmol each inhibitor). *Ep ac derm*- epidermal acantholytic dermatosis (the loss of intercellular connections, such as desmosomes, resulting in loss of cohesion between keratinocytes), *derm ed*- dermal oedema around skin annexes (here around the hair bulb), *necr*- necrotic changes around oedema, *vasc*- neovascularisation, *fib*- fibroblasts, *vess*- slightly dilated blood vessels due to acute inflammation, *neutr*- infiltrate enriched in neutrophils due to acute inflammation, *grt*- delayed maturation of granulation tissue, *fib at*- increased number of fibroblasts present in adipose tissue in miR-146a-5p inhibition treatment, *fib/mcr*- an influx of immune cells (fibroblasts and macrophages are predominant), most visible in combination treatments. Scale bars 50μm, magnification 400x with immersion oil (n=5 animals per group, 10 images taken per sample).

ESM Fig. 5

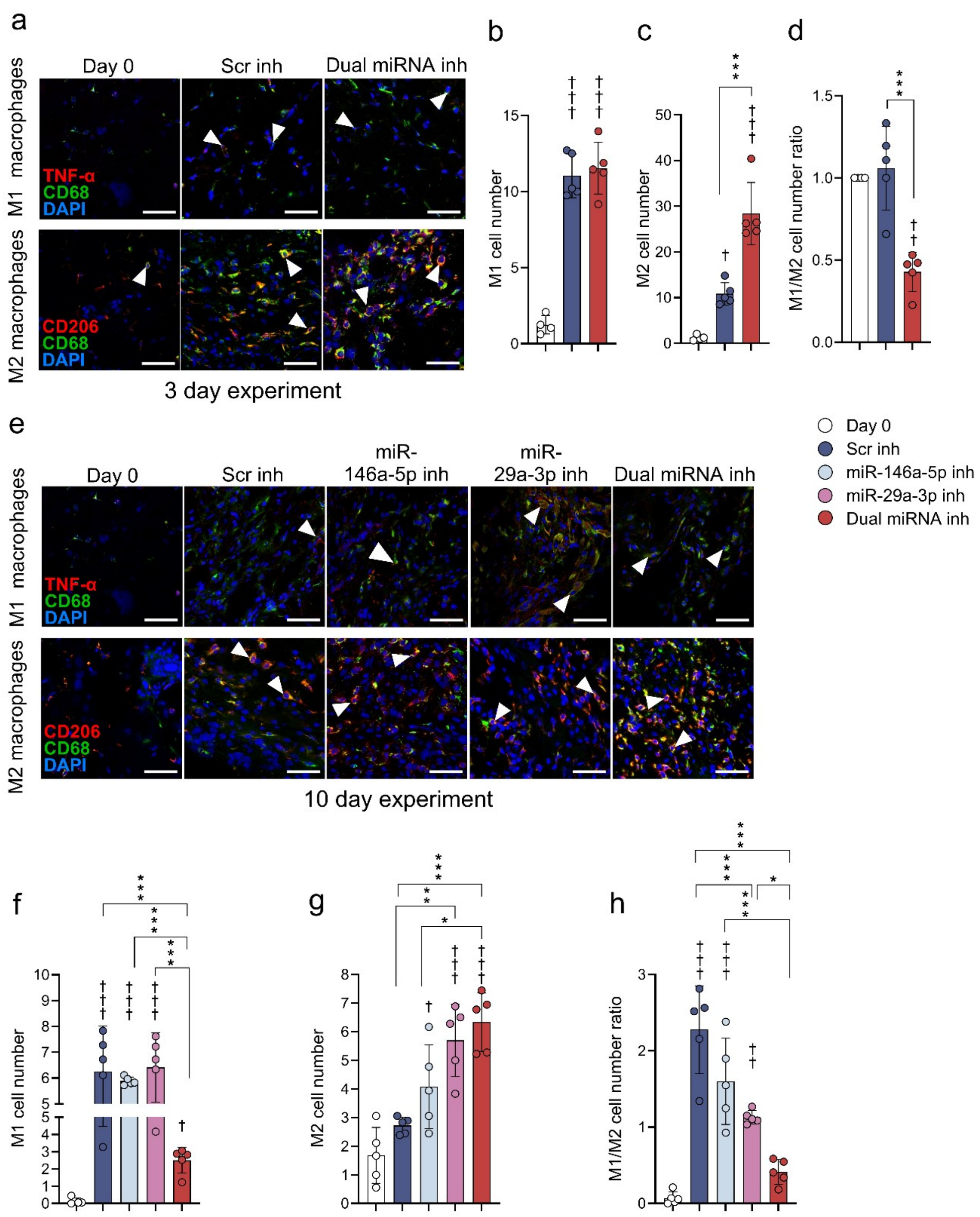

ESM Fig.5 Topical treatment with miR-46a-5p and miR-29a-3p inhibitors favours an anti-inflammatory wound environment. (a)-(h) Immunofluorescent analysis of the M1 and M2 macrophages in diabetic wounds (n=5), treated with Scr, miR-146a-5p/-29a-3p inhibitors individually or, the combination. (a) Representative fluorescent images of skin sections were obtained 3 days post-wounding (pro-inflammatory on the top and anti-inflammatory on the

bottom panel) (b) The number of pro-inflammatory macrophages quantified 3 days post-wounding and shown as the average number of CD68 positive cells co-stained with TNF-α. (c) Anti-inflammatory macrophages detected as cells stained with CD68 and co-stained with CD206. (d) The M1 to M2 ratio in skin sections 3 days post-surgical collection of skin. (e) Representative images from confocal microscopy showing the M1 (top panel) and M2 (bottom panel) macrophages in skin sections collected 10 days post wounding, tagged with paired CD68/TNF-α or CD68/CD206 antibodies (f) the number of pro-inflammatory macrophages quantified 10 days post-wounding (g) the number of anti-inflammatory macrophages quantified 10 days post-wounding (h) the M1 to M2 proportion as the quantitative assessment of the pro- and anti-inflammatory environment in the skin.
Cell abundance was counted per area using ImageJ2 software (n=5 animals/group, wounds in duplicates and 3 selected areas/ 6 pictures/sample. $^{*}p<0.05$, $^{**}p<0.01$, $^{***}p<0.001$ (miR-146a-5p/-29a-3p inhibition relative to Scr inhibition) and $^{\dagger}p<0.05$, $^{\dagger\dagger}p<0.01$, $^{\dagger\dagger\dagger}p<0.001$ (miR-146a-5p/-29a-3p inhibition/ Scr relative to d0 baseline values). Panel a, e: scale bars 50μm, magnification 400x with immersion oil.
